# Undernutrition and associated factors among children aged 6–59 months in nutrition-sensitive agriculture intervention implemented Basona district, North Shewa Zone, Amhara region, Ethiopia

**DOI:** 10.1371/journal.pone.0284682

**Published:** 2023-04-26

**Authors:** Gebretsadik Keleb Yehuala, Afework Hailu Orcho, Mizan Habtemichael Gebresilassie, Habtemariam Abate Meshesha, Tewodros Getnet Amera, Eshetu Zerihun Tariku

**Affiliations:** 1 School of Public Health, College of Medicine and Health Sciences, Jijiga University, Jijiga, Ethiopia; 2 College of Health Sciences, Addis Ababa University, Addis Ababa, Ethiopia; 3 Addis Ababa Food and Drug Control Authority, Addis Ababa, Ethiopia; 4 Department of Epidemiology and Biostatistics, College of Medicine and Health Sciences, Dire Dawa University, Dire Dawa, Ethiopia; 5 Department of Public Health Nutrition, College of Medicine and Health Sciences, Arba Minch University, Arba Minch, Ethiopia; COMSATS University Islamabad - Lahore Campus, PAKISTAN

## Abstract

**Background:**

In Ethiopia, child malnutrition is a significant public health problem. To address the problem, Nutrition-Sensitive Agriculture (NSA) program was introduced. However, there is a paucity of evidence about the prevalence of child undernutrition in NSA-implemented districts. Therefore, this study aimed to assess the prevalence of undernutrition among children aged 6–59 months in NSA-implemented districts.

**Method:**

A community-based cross-sectional study was conducted by enrolling 422 children aged 6–59 months paired with their mothers. A systematic sampling technique was used to select respondents. Data were collected by Open Data Kit (ODK) data collection platform, and Stata version 16 was used for analysis. The multivariable logistic analysis model was fitted to assess the association between variables, and 95% CI was estimated to measure the strength of the association. The level of statistical significance was declared at a p-value of less than 0.05 in the multivariable model.

**Result:**

Overall, 406 respondents participated in the study, and a response rate of 96.2% was obtained. The prevalence of stunting, wasting, and underweight was 24.1% (95% CI: 19.9–28.4), 8.87% (95% CI: 6.3–12.1) and 19.95% (95% CI: 16.2–24.2), respectively. Household food insecurity was significantly associated with being underweight (AOR: 3.31, 95% CI (1.7–6.3). Child dietary diversity (AOR: 0.06, 95% CI: 0.01–0.48) and being a beneficiary of the NSA (AOR: 0.12, 95% CI: 0.02–0.96) program were associated with wasting. Lack of ANC visits and diarrhea in the past two weeks was associated with stunting and wasting, respectively.

**Conclusion:**

The prevalence of malnutrition was a moderate public health problem. Wasting was more prevalent than the recent national and Amhara region averages. However, the prevalence of stunting and underweight was lower than the national average and other studies conducted in Ethiopia. Healthcare providers should work to increase dietary diversity, ANC visits, and reduce diarrheal disease.

## Introduction

### Background

Malnutrition remains a global public health challenge, with an estimated 821 million people undernourished in 2017, far higher than the prevalence rate reported in 2016 when 804 million people were undernourished. Undernourishment appears to be persistent in all regions of Africa, which remains the continent with the highest prevalence of undernutrition, affecting more than 256 million people [1].

Undernutrition refers to insufficient intake of energy and nutrients to meet an individual’s needs to maintain good health [2, 3]. The three most common forms of undernutrition among children under the age of five are stunting, wasting, and underweight, which makes children more vulnerable to disease and death [2]. Stunting is defined as low height for age. It is the result of chronic or recurrent undernutrition. The second form of undernutrition is wasting, defined as low weight-for-height, which indicates recent and severe weight loss. Underweight, often known as low weight for age, is another manifestation of undernutrition. An underweight child could be wasted, stunted, or both [2].

According to evidence between 2000 and 2019, the number of stunted children under five years of age worldwide declined from 199 million to 144 million [4]. At the same time, the number of stunted children has increased from 22.4 million to 29.0 million in West and Central Africa [4]. According to the Food and Agricultural Organization (FAO) African region overview of nutrition in Sub-Saharan Africa, the prevalence of undernourishment appears to have risen from 20.8% to 22.7% between 2015 and 2016 [5]. In Sub-Saharan Africa, nearly all countries experience multiple burdens of malnutrition due to inadequate and unbalanced consumption of macro and micronutrients [5].

In Ethiopia, the prevalence of child stunting decreased by 14% (from 51% to 37%) between 2005 and 2019. During the same period, the percentage of wasting children reduced from 12% to 7%, while the number of underweight children decreased by 12% (from 33% to 21%) [6]. According to the Ethiopian Mini-Demographic and Health Survey (EDHS 2019), the prevalence of child stunting, underweight, and wasting was 37%, 21%, and 7%, respectively [6]. In Ethiopia, the factors most often associated with childhood stunting and undernutrition include low maternal education, short maternal stature, the late beginning of complementary feeding, food insecurity, and low household wealth index [7, 8].

Several interventions targeting the improvement of overall malnutrition have been implemented in Ethiopia [9]. Nutrition-Sensitive Agriculture (NSA) is a crucial recommendation for improving nutrition. Addressing the underlying causes of malnutrition contributes to long-term improvements in the nutritional and health status of the population. NSA, in particular, increases the availability, accessibility, and consumption of food that meets peoples’ nutritional requirements while minimizing unintended adverse nutritional effects on health and care. It alleviates all forms of malnutrition and, in the end, achieves healthy and sustainable diets [10]. According to FAO, NSA is defined as a food-based approach to agricultural development that puts nutritionally rich foods, dietary diversity, and food fortification at the heart of overcoming malnutrition with the primary objective of making food more accessible, diverse, nutritious, and sustainable in production [11].

Agricultural interventions increase household income through commercialization that enhances household food security, dietary diversity, and women’s empowerment, all of which are major associated factors of childhood undernutrition [10, 12]. Furthermore, agricultural interventions positively impact knowledge, attitude, practice, asset building, and overall poverty reduction, which increases household consumption of diversified foods [10, 12].

The National Nutrition Program II, as depicted in strategic objective two, and national nutrition policy with the vision "to attain optimal nutritional status at all stages of life span and conditions to a level that is consistent with good health, quality of life and productivity" emphasize nutrition-sensitive interventions as priority intervention strategies. Nutrition-sensitive interventions are priority interventions to reduce child undernutrition sustainably [13, 14].

To overcome the problem and achieve the strategic objective, Ethiopia is committed to achieving sustainable development goals as a member of the United Nations. The government strives to ensure access to safe, nutritious foods for all people all year round and eradicate all forms of malnutrition. The country has implemented some NSA interventions to improve the nutritional status of children and women by increasing the quantity and quality of available, accessible, and affordable food and promoting the utilization of diverse, nutritious, and safe foods for all Ethiopians at all times [12].

A systematic review of six countries determined that NSA interventions positively impacted intermediate nutrition outcomes on the pathway from agricultural intervention to nutritional or health status, including dietary diversity of households and individuals. However, there was no convincing evidence of the impact of agricultural interventions on child anthropometric measurement [15].

Evidence was limited about the prevalence of undernutrition and its associated factors in nutrition-sensitive agriculture-implemented districts. Studies also recommend frequent research on the prevalence of undernutrition to fully understand the trend in the prevalence of the problem in the county [16]. To improve the program implementation, the challenges faced so far, and the associated factors of undernutrition in those districts must be assessed, identified, and addressed accordingly.

Therefore, this study aims to assess the prevalence of undernutrition and associated factors among children aged 6–59 months in an NSA intervention implemented in Basona districts, North Shewa Zone, Ethiopia.

### Conceptual framework

**Fig 1:**
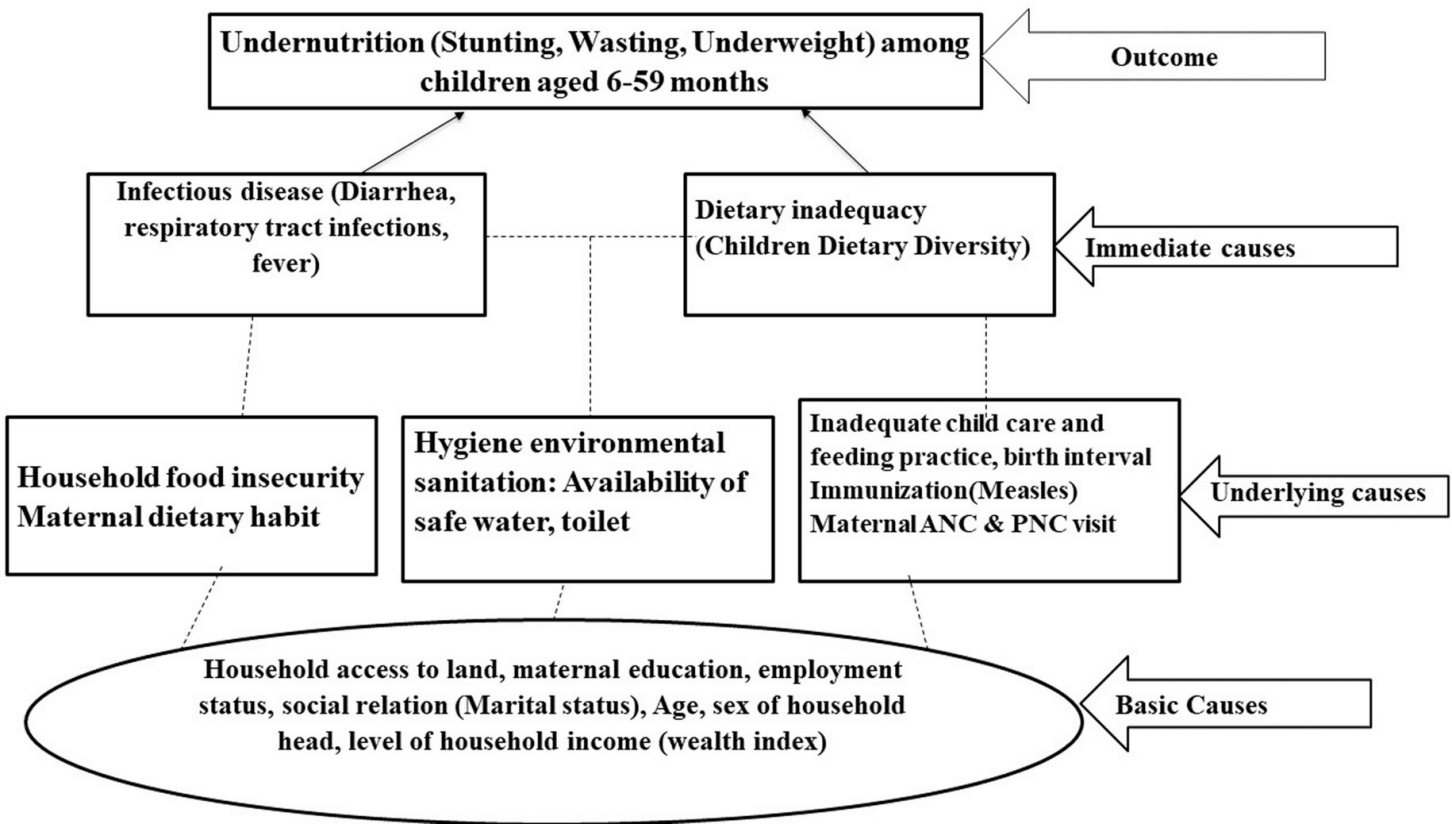
Conceptual framework for the cause of undernutrition Adapted from UNICEF 1991 (updated in 2021), for illustrative purposes only.

The conceptual framework was adapted from the UNICEF conceptual framework updated in 2021. The basic causes, such as the economic and social relations, and the underlying causes, namely household food security, availability of child and maternal care services, and hygiene and sanitary practices, are linked with each other, and the outcome variable (undernutrition) (Fig 1).

## Methods

### Study area

The study was conducted in North Shewa Zone, Amhara Regional State, in the randomly selected Bosena Worena district, which is located at 9° 49’ 59.99" N Latitude and 39° 19’ 60.00" E longitude, 130 km from the capital Addis Ababa and 695 km from the regional capital Bahir Dar. According to the population projection value of the 2017 Ethiopian Central Statistics Agency (CSA), the total population of North Shewa Zone was 2,248,418, from which about 1,114,301 were females. The projected total population in Basona Worena district in 2017 was 140,386 [17]. Almost all residents of the Basona Worena district are ethnic Amhara and Orthodox Christians. More than 95% of the population lives in rural areas and relies on agriculture.

### Study design and period

A community-based cross-sectional study was employed from May 2021- June 2021 to assess the prevalence of undernutrition among children aged 6–59 months in a randomly selected NSA intervention implemented Basona district. All mother-child pairs with a child aged 6–59 months who reside in Basona-Worena district (NSA intervention district) were the source population for this study. The study population was all systematically selected mother-child pairs who have a child aged 6–59 months selected from four randomly selected NSA intervention sub-districts at the time of data collection.

### Sample size determination and sampling procedure

The sample size for the first specific objective was determined using a formula for estimating a single population proportion.


$$n=\frac{{\left(Z\frac{\alpha }{2}\right)}^{2}P(1-P)}{d^{2}}$$

Where: *n* = the required sample size, *Z* = the value of Z in the standard normal distribution that corresponds to a level of 0.05, *p* = Assumed population proportion p = 50% and *d* = the margin of error (precision) = 5%

$$So,n=\frac{1.96^{2}(0.5(1-0.5))}{0.05^{2}}=384$$

Taking a 10% non-response rate, the final sample size becomes 422.

#### Sampling procedure

A simple random sampling technique was used to select NSA program implemented district in North Shewa Zone. Then, from the selected district (*Basona-Werana* district), eight sub-districts (Kebele in local terms) were included in the study. The sample size was proportionally allocated to each sub-district. In each sub-district, households with women having children 6–59 months of age (a pair of woman and child) were selected through a systematic sampling technique after a list of maternal-child pair data within the eligibility age range were obtained from each sub-district family folder. Households with more than one under-5 child were selected using the lottery method. Mother-child pairs with a child aged 6–59 months in the selected district were eligible for this study at the time of data collection. However, children whom the physician informed to avoid movement due to a fear of dislocation, severe pain during movement, or spinal cord injury were excluded from the study.

### Data collection tool

The data were collected using the Open Data Kit (ODK) data collection platform, an open-source electronic tools that allows data collection using mobile devices and submission to an online server and facilitate accurate and efficient data gathering.

The questionnaire was adapted from the EDHS household wealth index assessment tool, WHO infant and young child minimum dietary diversity assessment tool, and the Food and Nutrition Technical Assistant Program (FANTA) Household Food Insecurity Access Scale (HFIAS) assessment tool [18, 19]. The household wealth index assessment tool was adapted from the EDHS standard tool which is a survey-specific measure of the relative economic status of households.

The HFIAS, developed by USAID-funded FANTA project, is built on a short questionnaire that captures households’ behavioral and psychological manifestations of insecure food access, such as having to reduce the number of meals consumed or sacrificing food quality owing to a lack of resources [19]. The tool was validated in Ethiopia, and it was recommended that the tool can be adapted to various seasonal contexts in the country with due consideration of cultural and contextual factors [20]. A face-to-face interview was employed to fill out the prepared structured questionnaires. The questionnaire was prepared in English first and translated into Amharic (the local language); finally, it was back-translated to English for data consistency.

### Data collection procedure

Children anthropometric measurements, such as weight to the nearest 0.1 kg and height to the nearest 0.1 cm, were taken using a calibrated UNICEF weight scale and measuring board that was readily available in the area. Length was measured by a measuring board in a recumbent position for children less than age two years with the nearest 0.1 cm [21]. Data were recorded after three repeated measurements, and the average was used for analysis. Paired trained data collectors collected data with frequent monitoring by the principal investigator [22].

The HFIAS assessment tool was used to determine households’ level of food insecurity [18, 19]. HFIAS was assessed by asking whether, in the last thirty days, the household had ever worried about having enough food, had to reduce food intake because of shortages of food or lack of money to buy food, or had to go without having eaten because of the shortage of food or lack of money to buy food and had to ask outside the home for food because of a shortage of food or lack of money to buy foods.

WHO infant and young child minimum dietary diversity assessment tools were used for assessing children minimum dietary diversity. Child dietary diversity was assessed by interviewing parents or caregivers. The interviewer asked about the different types of foods the child ate the day before the interview, using a 24-hour recall approach. The indicator is based on the count of 7-food groups before the actual date of data collection. Food groups include grains, roots, and tubers; legumes and nuts; dairy products (milk, yoghurt, cheese); flesh foods (meat, fish, poultry, and liver/organ meats); eggs; vitamin-A rich fruits and vegetables and other fruits and vegetables. Structured 24-hour recall of mothers was used to assess the foods consumed by the children. Finally, if the child consumed four or more food groups, they were considered to have adequate dietary diversity [23].

The household wealth index was determined and analyzed using principal component analysis by considering household assets such as livestock, type of house, and durable, productive assets. All the variables were changed into binary variables into "1" if the variable(asset) exists and "0" if the variable does not exist. First, the frequency of each variable was calculated. Variables with frequencies of less than 5% and greater than 95% were excluded from the principal component analysis because they do not help distinguish households by wealth index. Next, the scale was divided into five quantiles: the lowest quantile representing poverty and the highest quantile representing wealth [24]. The outcome variable for this study was undernutrition among children 6–59 months (stunting, underweight and wasting).

### Operational definitions

S1 Appendix.

### Data quality assurance

To ensure the completeness, accuracy and consistency of data collection, the data were collected by data collectors with bachelor’s degrees in nursing and public health. The data collectors were recruited from the same cultural background and have worked in the area for a long time. Two days of training were given to data collectors. A meeting session was held every day of the data collection period and thorough checking was done before using the questionnaires. A pretest on 24 maternal-child pairs was conducted in *Angolala Tera* Woreda. After the pretest, the ordering of questions, the interviewing techniques, and the estimated time needed for the interview were estimated and arranged accordingly. TEM was determined to assess intra and inter-measurer reliability [25].

### Data management and statistical analysis

Data were collected by using ODK, exported and analyzed using STATA version 16, and anthropometric indices were calculated by WHO-Anthro and interpreted according to WHO growth standards. Descriptive statistical summary measures were used to describe variables. Binary logistic regression analysis with an odds ratio and a 95% confidence interval was used to assess the degree of association between variables. Variables with a p-value <0.25 were entered into a multivariable analysis model [26].

Model goodness of fit was determined using the Hosmer-Lemeshow statistical test of goodness of fit. Adjusted odds ratio (AOR) was applied to identify associated factors for the prevalence of child undernutrition and used to control for possible confounding effects. The final finding was reported using crude odds ratio (COR) and adjusted odds ratio (AOR) along with 95% CI. Statistical significance was declared at a p-value of less than 0.05 in the multivariable model.

### Ethical considerations

Ethical approval to conduct the study was obtained from Kotebe Metropolitan University ethical review board. Permission was obtained from North Shewa Zone health office and Basona district health bureau. For literate mothers, a written information sheet was distributed. The data collectors read the information sheet to mothers who cannot read or write. Unclear questions were clarified. The information sheet includes a detailed explanation of the survey’s purpose, a description of the benefits, and the absence of potential risks. They were also informed that they could withdraw their consent and end the interview at any time. No personal names were used as an identifier. The collected data was kept confidential, and no one had access to the collected data except the research team. Finally, written consent was obtained from mothers/caregivers. Children with an apparent manifestation of severe wasting were linked to the nearby health center.

## Results

### Socio-demographic characteristics of study respondents

A total of 406 respondents participated in the survey, and a response rate of 96% was obtained. More than half of the study respondents were females (53.45%), while the ages of children ranged from 12 to 23 months. The mean(±SD) age of respondent mothers was 31.1 (±6.37) years. The majority of the study respondents (96.55%) were rural dwellers. The primary source of drinking water for the assessed households was piped water 367 (90.39%). Regarding the beneficiary of NSA, only 106 (26.11%) households were beneficiaries of the program, among which 55 (58.51%) households benefited from the program for three years and above (Table 1).

**Table 1:** Socio-demographic and economic characteristics of respondents at Basona district, North Shewa Zone, Ethiopia, June 2021 (N = 406).

Variable	Category	Frequency (N)	Percentage (%)
**Maternal age (in years)**	15–24	58	14.29
	25–34	226	55.67
	35–44	106	26.11
	44+	16	3.94
**Place of residence**	Rural	14	3.45
	Urban	392	96.55
**Maternal marital status**	Single	15	3.69
	Married	359	88.42
	Other*	32	7.88
**Maternal level of education**	No formal education	241	59.65
	Formal education	163	40.35
**Father level of education**	No formal education	236	58.13
	Primary school	120	29.56
	Secondary school and above	50	12.32
**Maternal occupation**	Housewife	373	91.87
	Farmer	11	2.71
	Government employee	7	1.72
	Daily laborer	6	1.48
	Student	9	2.22
**Head of household**	Wife	47	11.58
	Husband	359	88.42
**Sex of the child**	Male	187	46.55
	Female	217	53.45
**Age of the child (in months)**	6–11	36	20.20
	12–23	119	19.92
	24–35	99	19.95
	36–47	82	19.95
	48–59	70	19.95
**Family size (in number)**	<5	266	65.52
	5+	140	34.48
**Number of under-five children**	1	168	41.38
	2	228	56.16
	3+	10	2.46
**Type of latrine is available**	Pit latrine with slab	64	15.76
	Pit latrine without a slab	249	61.33
	No latrine/bush/field	93	22.29
**Source of drinking water**	Piped water	367	90.39
	Protected spring	29	7.14
	Unprotected spring	10	2.46
**Have a separate kitchen**	Yes	299	73.65
	No	107	26.35
**Beneficiary of NSA program**	Yes	106	26.11
	No	300	73.89
**Have agricultural land**	Yes	281	69.90
	No	121	30.10
**Wealth quantile**	Poorest	82	20.20
	Poor	81	19.92
	Middle	81	19.95
	Rich	81	19.95
	Richest	81	19.95

*- Divorced/Separated

### Household food insecurity

**Fig 2:**
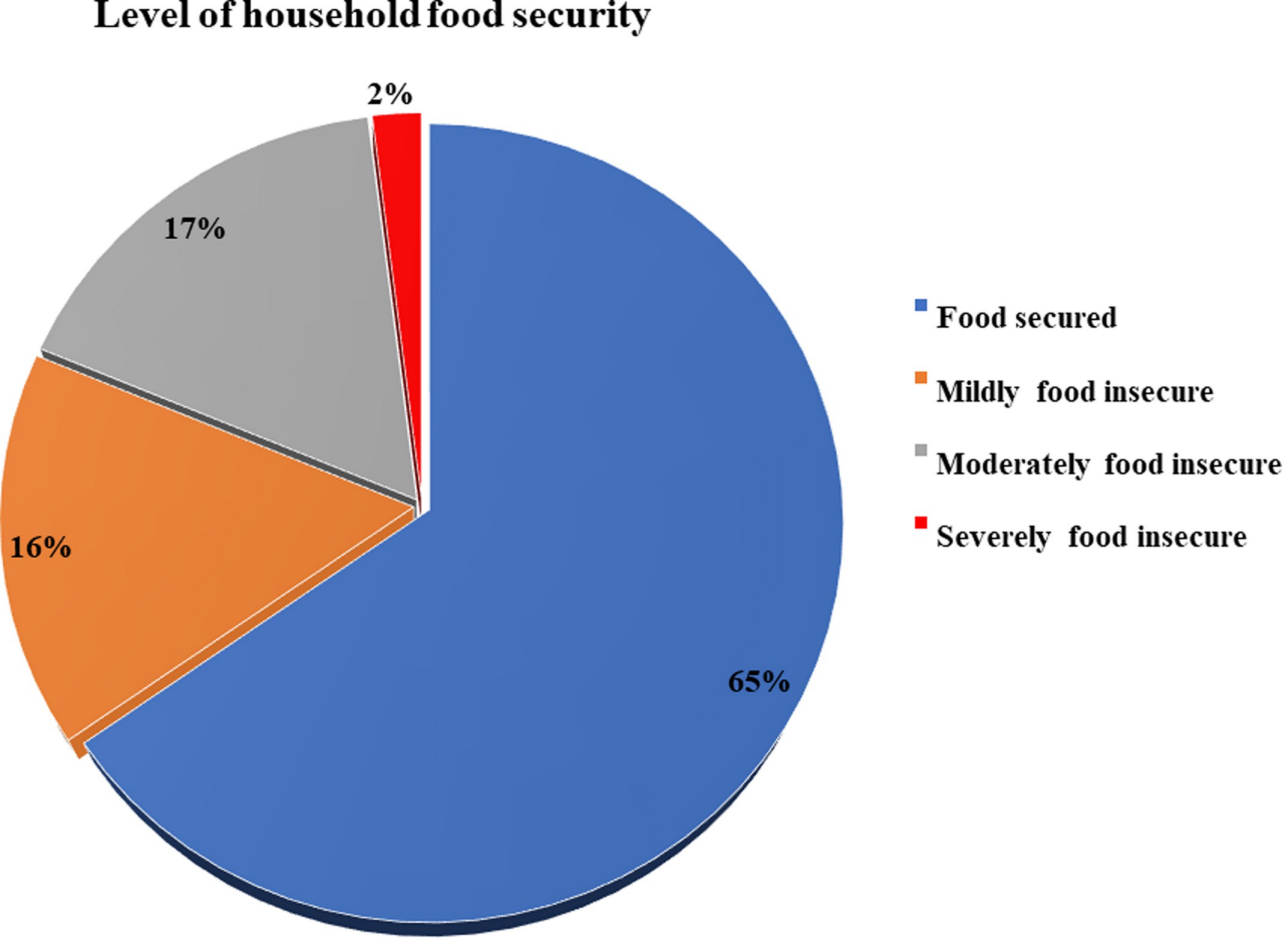
Prevalence of household food insecurity in Basona Worena district, North Shewa Zone, June 2021.

Households who experienced worry and uncertainty about food availability in the four weeks preceding data collection were 155 (38.18%). About 117(28.82%) of households were unable to eat the kind of food they preferred because of a lack of resources, 114 (28.08%) and 45 (11.08%) of households eat a limited variety of foods and eat some foods that they did not want to at because of a lack of resources to obtain other food, respectively. Only eight (1.97%) and three (0.74%) household members sleep hungry at night and stay a whole day and night without eating anything because there were food shortages in the four weeks prior to data collection, respectively. Regarding the coping mechanism during food insecurity and shortage of food items in the household, 304 (76.38%) households mentioned they would sell household properties, furniture and cattle to cope with the problem. About half, 55.03% or 219 households, mentioned they would increase their income by doing more work, 248 (62.31%) mentioned they will reduce the cost of non-food items, and 57 (14.32%) mentioned they would receive financial and in-kind food items from NGOs and government offices as a coping mechanism during the shortage of food items. The overall prevalence of food insecurity in the assessed households was 141(34.73%) (Fig 2).

### Infant and Young Child Dietary Diversity (IYCDD)

**Fig 3:**
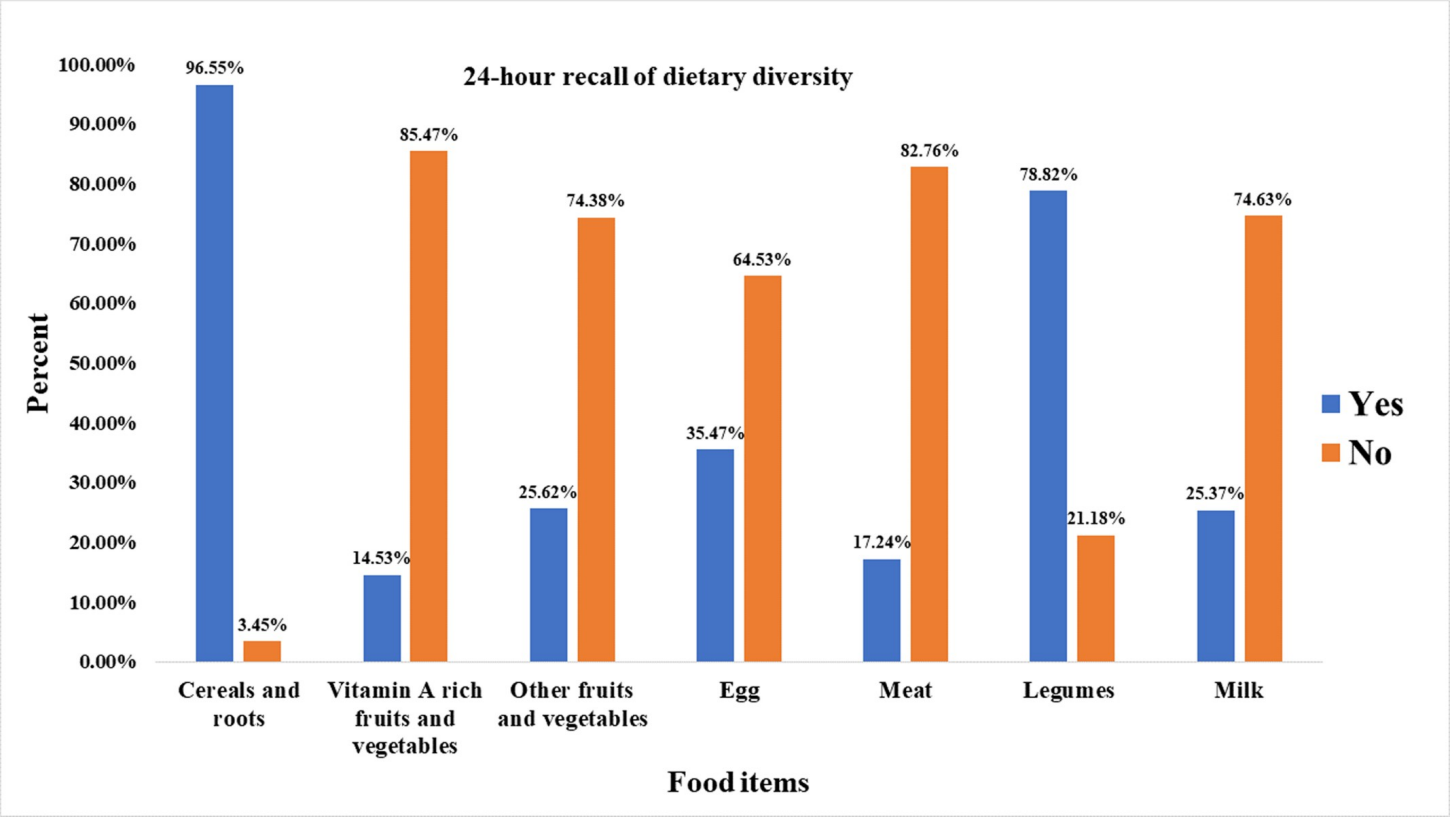
Infant and young child dietary diversity among children aged 6–59 months in Basona district, North Shewa Zone, June 2021.

The most commonly consumed food items by children were cereals and roots 392 (96.55%), followed by legumes and nuts 320 (78.82%). Only 70 (17.24%) children consumed any type of meat, including organ meats. No children consumed fish and seafood in the past 24 hours before the actual time of the interview. Generally, only 109 (26.85%) children consumed four or more food groups and were categorized as receiving an adequate dietary diversity score (Fig 3).

### Prevalence of undernutrition among children aged 6–59 months

**Fig 4:**
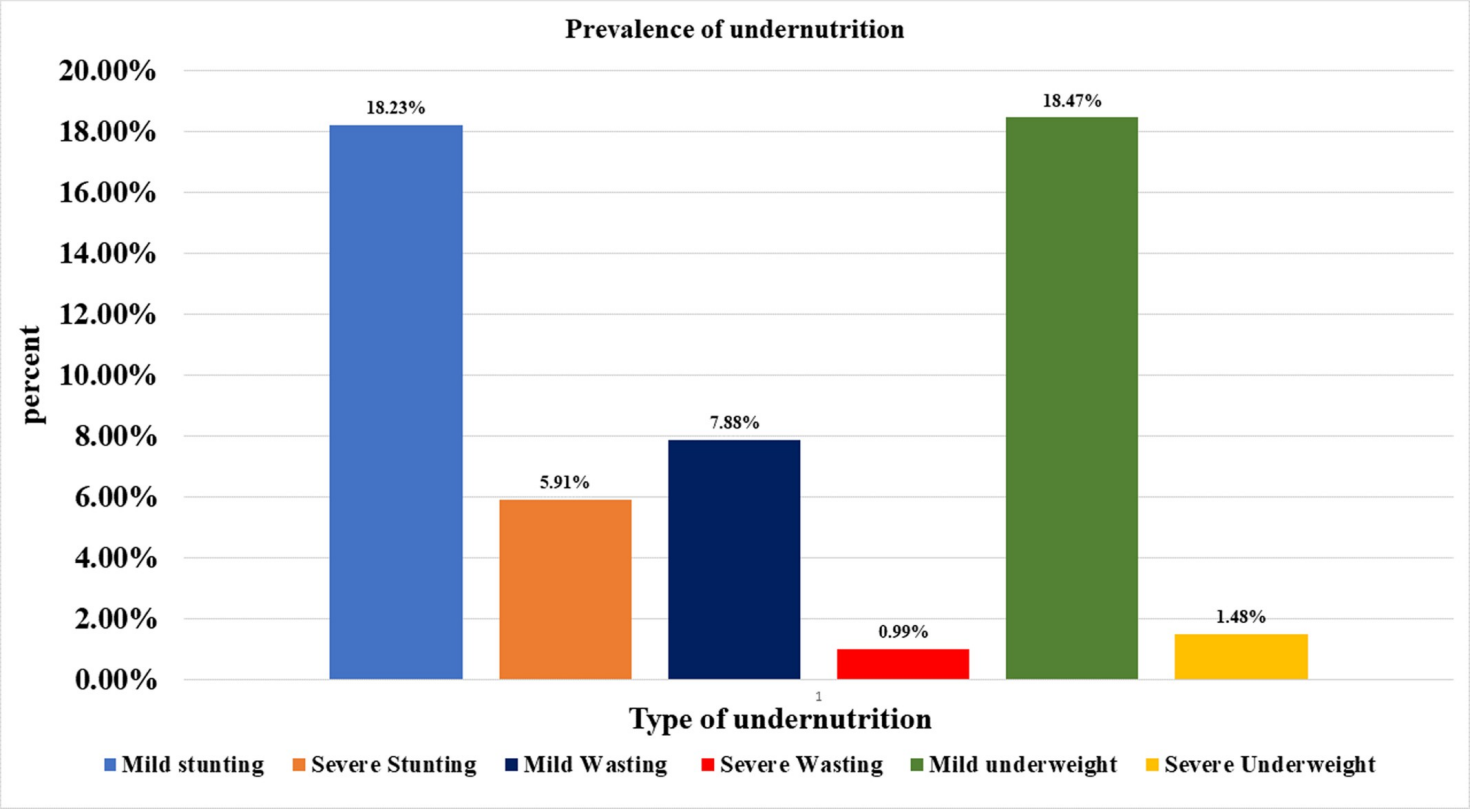
Prevalence of undernutrition among children aged 6–59 months in Baso district, North Shewa Zone, June 2021.

The prevalence of stunting, wasting and underweight in the study area was 24.1% (95% CI = 19.92–28.48), 8.87% (95% CI = 6.29–12.06) and 19.95% (95% CI = 16.17–24.17), respectively. Among all, male children 55 (29.10%) were stunted, while 42 or 19.3% of females were stunted. An almost equal percentage of males and females were wasted (8.99% and 8.76%), respectively (Fig 4).

### Factors associated with undernutrition

#### Factors associated with stunting

In a multivariable analysis, children born from mothers who gave their first birth at 20 years and above were 60% [(AOR = 0.40, 95% CI: (0.21,0.73)] less likely to be stunted than those children who were born from mothers who gave first birth before 20 years of age. Children from the wealthiest wealth index family were 98% [(AOR = 0.02, 95% CI: (0.003,0.13)] less likely to be stunted than children from the poorest wealth index households. Children aged 36–47 months and aged 48–59 months were 6.41 [(AOR = 6.41, 95% CI: (1.60,25.67)] and 5.75 (AOR = 5.75, 95% CI: (1.38,23.99)] times more likely to be stunted than those aged 6–12 months respectively (Table 2).

**Table 2:** Bivariable and multivariable analysis of factors associated with stunting among children aged 6–59 months in Basona district, North Shewa Zone, June 2021 (N = 404).

Variables	Stunting		COR (95% CI)	AOR (95% CI)
	Yes (N) %)	No (N) %)		
**Maternal age group (year)**				
15–24	15 (25.86)	43 (74.14)	1	1
25–34	51 (22.57)	175 (77.43)	0.84 (0.43–1.63)	0.88 (0.38–2.03)
35–44	24 (23.08)	80 (76.92)	0.86 (0.41–1.81)	1.16 (0.41–3.32)
>44	7 (43.75)	9 (56.25)	2.23 (0.71–7.04) *	3.78 (0.58–24.87)
**Maternal age at first birth**				
<20 years	68 (30.63)	154 (69.37)	1	1
>20 years	29 (16.02)	152 (83.98)	0.43 (0.26–0.70) **	0.40 (0.21–0.73) **
**Number of under-five children**				
1	35 (20.83)	133 (79.17)	1	1
2	59 (26.11)	167 (73.89)	1.34 (0.83–2.16) *	1.58 (0.85–2.96)
3	3 (30)	7 (70)	1.62 (0.40–6.62)	0.77 (0.13–4.53)
**Total family size**				
<5	54 (20.38)	211 (79.62)	1	1
5+	43 (30.94)	96 (69.06)	1.75 (1.10–2.79) *	2.72 (1.28–5.80) **
**House ownership**				
Private	87 (23.90)	277 (76.10)	1	1
Rent & others	10 (25.00)	30 (75.00)	1.06 (0.50–2.26) *	0.60 (0.24–1.51)
**Household wealth index**				
Poorest	24 (29.27)	58 (70.73)	1	1
Poor	26 (32.10)	55 (67.90)	1.14 (0.59–2.22)	1.23 (0.52–2.94)
Middle	28 (34.57)	53 (65.43)	1.28 (0.66–2.47)	0.86 (0.33–2.06)
Rich	17 (20.99)	64 (79.01)	0.64 (0.31–1.31)	0.68 (0.23–2.01)
Richest	2 (2.53)	77 (97.47)	0.06 (0.01–0.28) **	0.02 (0.003–0.13) **
**Sex of the child**				
Male	55 (29.1)	134 (70.90)	1	1
Female	42 (19.53)	173 (80.47)	0.59 (0.37–0.93) * *	0.56 (0.30–1.02)
**Food Insecurity category**				
In-secure	39 (27.66)	102 (72.34)	1.35 (0.84–2.16)	0.85 (0.46–1.56)
Secure	58 (22.05)	205 (77.95)	1	1
**Age of the child in months**				
6–11	3 (8.33)	33 (91.67)	1	1
12–23	23 (19.66)	94 (80.34)	2.69 (0.76–9.55)	4.32 (1.10–16.97) *
24–35	24 (24.24)	75 (75.76)	3.52 (0.99–12.51) *	3.49 (0.87–13.97)
36–47	29 (35.37)	53 (64.63)	6.02 (1.70–21.34) *	6.41 (1.60–25.67) *
48–59	18 (25.71)	52 (74.29)	3.81 (1.04–13.94) *	5.75 (1.38–23.99) *
**Type of toilet facility**				
Pit latrine with slab	12 (18.75)	52 (81.25)	1	1
Pit latrine without slab	61 (24.70)	186 (75.30)	1.42 (0.71–2.84) *	2.26 (0.91–5.59)
No latrine/bush/field	24 (25.81)	69 (74.19)	1.51 (0.69–3.29)	2.00 (0.72–5.60)
**Own agricultural land**				
Yes	50 (17.92)	229 (82.08)	1	
No	47 (38.84)	74 (61.16)	2.91 (1.81–4.69) **	3.15 (1.55–6.41) **
**Presence of AURTI in the past two weeks**				
Yes	19 (23.46)	62 (76.54)	0.96 (0.54–1.71)	1
No	78 (24.15)	245 (75.85)	1	0.94 (0.44–2.04)
**Feeding frequency during pregnancy**				
Just like normal times	37 (28.46)	93 (71.54)	1	1
Increased	42 (19.53)	173 (80.47)	0.61 (0.37–1.01) *	0.85 (0.42–1.74)
Decreased	18 (30.51)	41 (69.49)	1.01 (0.56–2.16)	1.24 (0.52–2.95)
**Ever used FP**				
Yes	46 (20.63)	177 (79.37)	1	1
No	51 (28.18)	130 (71.82)	1.51 (0.95–2.39) *	1.03 (0.56–1.90)
**Mother received PNC visit**				
Yes	39 (19.60)	160 (80.40)	1	1
No	58 (28.29)	147 (71.71)	1.62 (1.02–2.57) *	1.49 (0.81–2.74)
**Mother received ANC visit**				
Yes	85 (23.04)	284 (76.96)	1	1
No	12 (34.29)	23 (65.71)	3.32 (1.70–6.48) **	4.49 (1.80–11.19) **
**Maternal height**				
≤1.50	24 (34.29)	46 (65.71)	1.87 (1.07–3.26) *	1.91 (0.85–4.28)
>150	73 (21.86)	261 (78.14)	1	1

* = significant at p< 0.05,

** = significant at p<0.01

*** = significant at p<0.001

AOR = Adjusted Odds Ratio, COR = Crude Odds Ratio

#### Factors associated with wasting

From the multivariable analysis, the odds of children from households who are beneficiaries of the NSA program were 88% [AOR = 0.12, 95% CI: (0.02,0.97)] less likely to be wasted than households who were not a part of the program. Besides, children who consume four and above four groups of food items from a total of seven food groups were 94% [AOR = 0.06, 95% CI: (0.01,0.48)] less likely to be wasted than those who consumed less than four food items in the past 24 hours before the date of data collection. Children who had diarrhea in the past two weeks before the date of data collection were 6.27 [AOR = 6.27, 95% CI: (2.59,15.15)] times more likely to be wasted than those who have no history of diarrhea in the past two weeks (Table 3).

**Table 3:** Bi-variable and multivariable analysis of factors associated with wasting among children aged 6–59 months in Basona district, North Shewa Zone, June 2021 (N = 406).

Variables	Wasting Frequency (%)		COR (95% CI)	AOR (95% CI)
	Yes	No		
**Paternal level of education**				
No formal education	18 (7.63)	218 (92.37)	1	1
Primary school	11 (9.17)	109 (90.83)	1.22 (0.56–2.67)	2.14 (0.72–6.40)
Secondary and above	7 (14)	43 (86)	1.97 (0.78–5.01) *	2.71 (0.77–9.60)
**Number of under five-child**				
1	21 (12.50)	147 (87.50)	1	1
2	14 (6.14)	214 (93.86)	0.46 (0.23–0.93) *	0.55 (0.23–1.31)
3	1 (10)	9 (90)	0.78 (0.09–6.45)	0.68 (0.07–6.71)
**Ownership of agricultural land**				
Yes	20 (7.12)	261 (92.88)	1	1
No	16 (13.22)	105 (86.78)	1.99 (0.99–3.98) *	1.32 (0.55–3.19)
**Being beneficiary of NSA**				
Yes	1 (0.94)	105 (99.06)	1.99 (0.99–3.99) **	0.12 (0.02–0.96) *
No	35 (11.67)	265 (88.33))	1	1
**Child dietary diversity**				
Not adequate <4	3 (11.45)	263 (88.55)	1	1
Adequate 4+	2 (1.83)	107 (98.17)	0.07 (0.01–0.51) **	0.06 (0.01–0.48) *
**Child age group**				
6–11	4 (11.11)	32(88.89)	1	1
12–23	5 (4.20)	114 (95.80)	0.35 (0.09–1.38) *	0.43 (0.09–2.10)
24–35	11 (11.11)	88 (88.89)	1 (0.10–3.37)	1.27 (0.30–5.30)
36–47	9 (10.98)	73 (89.02)	0.99 (0.28–3.44)	1.29 (0.29–5.69)
48–59	7 (10.00)	63 (90.00)	0.89 (0.24–3.26)	1.34 (0.28–6.40)
**Diarrhea in the past 2 weeks**				
Yes	15 (26.32)	42 (73.68)	5.58 (2.67–11.64) **	6.27 (2.59–15.15) **
No	21 (6.02)	328 (93.98)	1	1

* = significant at p< 0.05,

** = significant at p<0.01

*** = significant at p<0.001

#### Factors associated with underweight

From the multivariable logistic regression model, a maternal age of 35–44 years and above 44 years was significantly associated with the prevalence of underweight [AOR = 3.82, 95% CI: (1.15,12.67)] and [AOR = 17.04, 95% CI: (3.25,89.35)], respectively. Children in the age group above 35 months were also significantly associated with being underweight. In addition, children from mothers who decreased the consumption of additional food during pregnancy were 3.34 [AOR = 3.82, 95% CI: (1.64,8.91)] times more likely to be underweight than those who consume just like regular times. However, children from food-insecure households were 3.31 times [AOR = 3.31, 95% CI: (1.73,6.32)] more likely to be underweight than children from food-secured households (Table 4).

**Table 4:** Bivariable and multivariable analysis of factors associated with underweight among children aged 6–59 months in Basona district, North Shewa Zone, June 2021 (N = 406).

Variables	Underweight Frequency (%)		COR (95% CI)	AOR (95% CI)
	Yes	No		
**Maternal age in years**				
15–24	8 (13.79)	50 (86.21)	1	1.00
25–34	46 (20.35)	180 (79.50)	1.60 (0.71–3.60)	2.06 (0.75–5.66)
35–44	19 (17.92)	87 (82.08)	1.36 (0.56–3.34)	3.82 (1.15–12.67) *
>44	8 (50)	8 (50)	6.25 (1.82–21.4) **	17.04 (3.25–89.35) **
**Head of Household**				
Wife	13 (12.77)	34 (87.23)	1	1
Husband	68 (8.36)	291 (91.64)	0.61 (0.31–1.22)	2.15 (0.82–5.66)
**Wealth Quantile**				
Poorest	20 (24.39)	62 (75.61)	1	1
Poor	21 (25.93)	60 (74.07)	1.09 (0.53–2.20)	1.27 (0.52–3.12)
Middle	15 (18.52)	66 (81.48)	0.70 (0.33–1.50)	0.79 (0.29–2.10)
Rich	11 (13.58)	70 (86.42)	0.49 (0 .22–1.10)	1 (0.25–2.60)
Richest	14 (17.28)	67 (82.72)	0.30 (0.30–1.39)	0.99 (0.29–3.33)
**Ownership of agricultural land**				
Yes	45 (16.01)	236 (83.99)	1	1
No	36 (29.75)	85 (70.25)	2.22 (1.34–3.68) **	1.59 (0.75–3.36)
**Food security status**				
Insecure	44 (31.21)	97 (68.79)	2.80 (1.70–4.60) ***	3.31 (1.73–6.32) **
Secure	37 (13.96)	228 (86.04)	1	1
**Age of the child in months**				
6–11	4 (11.11)	32 (88.89)	1	1
12–23	9 (7.56)	110 (92.44)	0.65 (0.19–2.27)	1.33 (0.30–5.94)
24–35	26 (26.26)	73 (73.74)	2.85 (0.92–8.84)	7.99 (1.68–38.00) *
36–47	21 (25.61)	61 (74.39)	2.75 (0.87–8.71)	7.51 (1.51–37.38) **
48–59	21 (30)	49 (70.00)	3.43 (1.08–10.92) *	12.30 (2.32–65.75) **
**Child Birth order**				
First	18 (26.09)	51 (73.91)	1	1
2^nd^, 3^rd^ or 4^th^	52 (19.77)	211 (80.23)	0.70 (0.37–1.29)	0.54 (0.23–1.26)
Above 4^th^	11 (14.86)	63 (85.14)	0.49 (0.21–1.14)	0.30 (0.09–1.00)
**Diarrhea in the past two weeks**				
Yes	20 (35.09)	37 (64.91)	2.55 (1.39–4.69) **	1.27 (1.05–4.91) *
No	61 (17.48)	288 (82.52)	1	1
**Mother received ANC visit**				
Yes	68 (18.58)	298 (81.42)	1	1
No	13 (32.50)	27 (67.5)	2.11 (1.04–4.30) *	2.34 (0.98–5.58)
**Child immunized for measles**				
Yes	75 (19.79)	304(80.21)	1.42 (0.84–2.41)	1.49 (0.79–2.88)
No	6 (22.22)	21 (77.78)	1	1
**Feeding frequency/pregnancy**				
Just like normal times	28 (21.54)	102 (78.46)	1	1
Increased	29 (13.36)	188 (86.64)	0.56 (0.32–0.99) *	0.77 (0.38–1.59)
Decreased	24 (40.68)	35 (59.32)	2.50 (1.28–4.86) **	3.82 (1.64–8.91) **

* = significant at p< 0.05,

** = significant at p<0.01

*** = significant at p<0.001

AOR = Adjusted Odds Ratio, COR = Crude Odds Ratio

## Discussion

The prevalence of stunting in our study was lower than the national average in Ethiopia, where the prevalence of stunting in the 2019 mini-EDHS survey was (37%) [6], and from a study conducted in Adama, Oromia Region (44%) [27]. The higher prevalence in the 2019 mini-EDHS may be due to the non-availability of the program in every DHS-assessed district and the consistent trend in the reduction of undernutrition in Ethiopia. However, the finding of our study was consistent with a study conducted in Southeast Ethiopia, Jima Zone, where the prevalence of stunting was 25.4% [28].

The prevalence of stunting in our study was lower than a finding from a survey conducted in Nigeria [29] and Bangladesh [30]. The inconsistency may be due to the fact that our study was conducted on nutrition intervention districts which may contribute to the reduction of the problem. However, the prevalence was higher than in the community-based cross-sectional study conducted in rural Zambia that assessed the prevalence of undernutrition in NSA intervention-implemented districts, where the prevalence of stunting was 21% [31]. The high prevalence of stunting in our study may be due to the long duration of the implementation of NSA programs in Zambia, as available in literature which can contribute to the reduction of stunting [32].

The prevalence of wasting in our study was 8.87% which is higher than the finding from the 2019 national average in which the prevalence was 7% [6]. The increase in the prevalence in our study may be due to the data collected during the pre-rainy seasons may contribute to the increase in diarrheal diseases. During pre-rainy dry seasons, the prevalence of wasting may be increased as justified by evidence [33]. The prevalence of wasting was higher than in the study from Zambia which aims to assess the association between NSA program and undernutrition, the prevalence in the study was 2% [31]. The inconsistency may be attributed to the reduction in household food items and the justified, proper integration of nutrition and agricultural programs in Zambia [32]. The result signifies that the appropriate integration of child nutrition and agricultural intervention pathways needs strict monitoring and evaluation to reduce child undernutrition.

The prevalence of underweight in our study was 19.5%, consistent with national averages from 2019 [6] and a study from Alefa District, North Gondar [34]. However, the prevalence was nearly twice that of a study from one of NSA implemented districts in Zambia, where the prevalence of underweight was 9.5% [31]. The discrepancy in the finding may be attributed to the length of the program and the variety of agricultural products being cultivated. In our study setup, the production of diversified agricultural products were focused on cereals and roots and livestock production activities. This implies that the NSA focusing on the production of diversified agricultural products can contribute to the reduction of underweight. However, the result was consistent with the study conducted in Northwest Ethiopia [34].

Children from households who are in the richest wealth quantile were less likely to be stunted than the poorest wealth quantile households. The finding was consistent with the study conducted in Wollayta Sodo, Southern Ethiopia, in which being stunted was 78% less likely in the richest wealth quintile households [35], Dabat Woreda, Northern Ethiopia [36] and with the evidence from the 2016 demographic survey [37], the result may be due to poor households are less likely to afford for diversified diets and lack of coping mechanisms in a difficult time that may contribute to the increase in the prevalence of stunting. In this study, old maternal age was associated with an increase in the prevalence of stunting. The result was consistent with the study in Ethiopia [38], but it was inconsistent with the study conducted in India [39]; the possible reason for the inconsistency may be due to the tendency of Ethiopian mothers to have too much number children in the late ages than the young ones, which results in less attention and care given to the young child.

In this study, the prevalence of stunting was predicted by the child’s age, as those in the older age group were more likely to be stunted than young children. The finding was supported by the study conducted in Jima zone, Southern Ethiopia [28], and by a study from Hawasa city where under-five children with older age (48–59 months) had a higher chance of being stunted than the young age groups [40]. This might be attributed to the increased risk of diarrheal disease during complete cessation of breastfeeding as well as the poor feeding practice of months/caregivers when they provide exclusive complementary feeding for late child age groups. Children from mothers who had no ANC visit during pregnancy were more likely to be stunted than those who had ANC visits; the finding of the study was supported by the study conducted in Nepal [41]. The most likely explanation for this is that when mothers receive ANC follow-up, children are less likely to have intrauterine growth restrictions, and the mother will receive counselling services regarding her nutrition, which ultimately will affect the growth of children in the latter childhood ages.

Being a beneficiary of the NSA program in the district was significantly associated with the prevalence of wasting in which children who are from NSA beneficiary households were 0.12 times more likely to be stunted than the non-beneficiaries. The result signifies that those agricultural interventions have a promising effect in the reduction of wasting, which implies that integrated NSA programs that target both the improvement in nutrition status and different agricultural products to increase household income need to be strengthened. Children who consumed four and above of the seven food groups in the past 24 hours from the data collection were also less likely to be wasted than their counterparts. The possible explanation for this includes having good child dietary diversity contributes to nutritional adequacy, so it can ultimately improve wasting in children. The increase in child dietary diversity is associated with being a beneficiary of NSA program. So, the NSA program directly or indirectly contributes to the district’s wasting prevalence.

The other finding in the study was that children who had a history of diarrhea in the past two weeks before the data collection were more likely to be wasted than those with no diarrhea. The finding was consistent with the study from Bule Hora district, where diarrhea in the past two weeks was 1.5 more likely to be wasted than those without diarrhea [42].

In this study, household food security was significantly associated with the prevalence of childhood underweight. The finding was consistent with the survey from Sekela district, Western Ethiopia, in which the children from food-insecure households were 2.25 times more likely to be underweight than children from the food-secured households [43] and Haramaya district, where the risk of being underweight was 2.48 times more likely for food in secured households [44]. The most plausible justification for the decrease in the prevalence of underweight in food-secured households may be due to the availability of food items, and the absence of worry and uncertainty in the households can contribute to the reduction of underweight. The presence of diarrhea in the past two weeks before the date of data collection was associated with the prevalence of underweight, the finding was supported by the study from Bule Hora district, southern Ethiopia [42].

In our study, mothers aged above 35 years were associated with the prevalence of being underweight. The finding contradicted the study conducted in Northwest Ethiopia [34]. Mothers who decreased their feeding frequency during pregnancy were negatively associated with the prevalence of being underweight. The scientific evidence may support this that consuming additional meals during pregnancy can help meet the energy and calorie demand of children in their later lives and will reduce children from being underweight.

A limitation of the study, mainly the dietary diversity assessment, may be affected by recall bias. To avoid recall bias, we use different rehearsal mechanisms, such as the type of food item the child consumed during lunch, dinner, and morning time specifically. In addition, food insecurity assessment questions might be affected by social desirability bias, so to avoid social desirability bias, respondents were interviewed in a separate place after they were informed about the aim of the study.

## Conclusion

In conclusion, child undernutrition in the study area was a moderate public health problem. The prevalence of wasting was higher than the national average conducted in 2019 mini-EDHS data. However, the prevalence of stunting and being underweight was lower than other studies and the national average.

Diarrhea in the past two weeks before the data collection was associated with the prevalence of wasting and being underweight but not with stunting. Moreover, household food security was associated with the prevalence of children underweight but not with stunting and wasting. Being a beneficiary of the NSA program was found to reduce the prevalence of wasting, which signifies that NSA interventions have a promising effect in addressing wasting and improving child dietary diversity scores. Advanced maternal age was also associated with the prevalence of wasting and stunting.

This study found that children born to mothers who received ANC visits were less likely to be stunted than those without ANC visits. However, the status of PNC visits was not associated with any undernutrition types. In addition, this study signifies that child from mothers who decreased their feeding frequency compared to the regular times was more likely to be underweight than those who consumed just like the regular times.

Based on the finding of this study, we suggest the following recommendations.

1. MOH: Should strengthen nutritional intervention programs. Specifically, they should focus on integrating nutritional intervention programs and agricultural diversification programs with the concerned agricultural development office since NSA have a promising effect in reducing wasting.
2. Health center and health posts: Should raise communities’ awareness about the importance of ANC visits, diarrheal disease prevention, and the consumption of additional meals during pregnancy by using different behavioral change communication strategies.
3. Researchers: This research was community-based cross-sectional research which may have its limitation; we recommend researchers conduct a quasi-experimental or experimental study to identify the association between the NSA program and child undernutrition.

## Supporting information

S1 Appendix Operational definition. (DOCX)

S1 Dataset (DTA)

S1 Table Questionnaire. (DOCX)

S1 File (PDF)

S2 File (PDF)

## Abbreviations

EDHS: Ethiopian Demographic and Health Survey
FANTA: Food and Nutrition Technical Assistant Program
FAO: Food and Agricultural Organization
HAZ: Height for Age Z-score
HFIAS: Household Food Insecurity Access Scale
IYCDD: Infant and Young Child Dietary Diversity
NSA: Nutrition-Sensitive Agriculture
ODK: Open Data Kit
WAZ: Weight for Age Z-score
WHO: World Health Organization
