# Supplementary material for: Undernutrition and associated factors among children aged 6–59 months in nutrition-sensitive agriculture intervention implemented Basona district, North Shewa Zone, Amhara region, Ethiopia
Source: PLoS One. 2023 Apr 26;18(4):e0284682. doi: 10.1371/journal.pone.0284682 (PMC10132697; doi:10.1371/journal.pone.0284682)
Supplement: S1 Appendix — (DOCX) [file pone.0284682.s001.docx]

# Operational definitions

**Undernutrition:** In this study, nutritional status includes different forms of undernutrition (stunting, wasting, underweight) among children aged 6 -59 months.

**Stunting: Assessed by height/length for age**

Children whose height (length) for age Z-score is below minus two standard deviations (-2 SD) from the median of the reference population were considered short for their age (stunted). The Z score below minus three standard deviations (-3SD) from the median were considered severely stunted (1).

**Underweight: Assessed by weight for age**

Children whose weight for age Z score is below minus two standard deviations (-2 SD) from the median of the reference population were classified as underweight, and the Z score below minus three standard deviations (-3SD) from the median were considered severely underweight (1).

**Wasting: Assessed by weight for height**

Children whose weight for height Z score is below minus two standard deviations (-2 SD) from the median of the reference population were classified as wasted, and the Z score below minus three standard deviations (-3SD) from the median were considered severely wasted (1).

**Adequate minimum child dietary diversity**: It was measured by asking about the child's consumption from the seven food groups. It was collected based on a 24-hour recall method. A child who consumed four of the seven food groups was considered to have an adequate minimum dietary diversity score (2).

**Not adequate minimum child dietary diversity**: A child who consumed less than four of the seven food groups was categorized as not having an adequate minimum dietary diversity score (2).

**Food secured:** A food-secure household experiences none of the food insecurity (access) conditions, or just experiences worry, but rarely (3).

**Mild Food insecurity**: The household worries about not having enough food sometimes or often, and/or is unable to eat preferred foods, and/or eats a more monotonous diet than desired and/or some foods considered undesirable, but only rarely. But it does not cut back on quantity nor experience any of the three most severe conditions (running out of food, going to bed hungry, or going a whole day and night without eating (3).

**Moderate food insecurity:** A moderate food-insecure household sacrifices quality more frequently by eating a monotonous diet or undesirable foods sometimes or often, and/or has started to cut back on quantity by reducing the size of meals or number of meals, rarely or sometimes. But it does not experience any of the three most severe conditions (3).

**Severe food insecurity**: A severe food-insecure household has experienced to cutting back on meal size or the number of meals often, and/or experiences any of the three most severe conditions (running out of food, going to bed hungry, or going a whole day and night without eating), even as infrequently as rarely. In other words, any household that experiences one of these three conditions even once in the last four weeks (30 days) was considered severely food insecure (3).

**Beneficiary of NSA:** Households were considered beneficiaries of NSA intervention programs if they lived in governmentally recognized NSA intervention districts and received support from governmental and non-governmental organizations.

**Non-beneficiary of NSA:** If households are not currently beneficiaries of the NSA program but live in NSA implemented districts and are using an older form of agricultural activities, were considered non-beneficiaries of NSA program.

# References

1. World Health Organization. WHO Child Growth Standards: length/height-for-age, weight-for-age, weight-for-length, weight-for- height and body mass index-for-age : methods and development. Geneva; 2006.

2. World Health Organization. Indicators for assessing infant and young child feeding practices [Internet]. World Health Organization, editor. Geneva; 2010. Available from: http://www.who.int

3. Coates J, Swindale a, Bilinsky P. Household Food Insecurity Access Scale (HFIAS) for measurement of food access: indicator guide. Washington, DC Food Nutr Tech …. 2007;(August):Version 3.
