# Supplementary material for: Undernutrition and associated factors among children aged 6–59 months in nutrition-sensitive agriculture intervention implemented Basona district, North Shewa Zone, Amhara region, Ethiopia
Source: PLoS One. 2023 Apr 26;18(4):e0284682. doi: 10.1371/journal.pone.0284682 (PMC10132697; doi:10.1371/journal.pone.0284682)
Supplement: S1 Table — (DOCX) [file pone.0284682.s003.docx]

**English Version questioner**

| Date of interview: | | | | | | | | | | |
| --- | --- | --- | --- | --- | --- | --- | --- | --- | --- | --- |
| Questionnaire ID: | | | | | | | | | | |
| Name of data collector: | | | | | | | | | | |
| Date of appointment for next visit if respondent not available during first visit: | | | | | | | | | | |
| **Section 1: General socio-demographic characteristics of mother and household** | | | | | | | | | | |
| No | Questions | Responses (alternative choices) | | | | | | Code | | Skip |
| 101 | What is the age of mother in years | __________ years | | | | | |  | |  |
| 102 | What is your place of residence? | 1. Urban 2. Rural | | | | | |  | |  |
| 103 | \| What is your current marital status? \|  \| \| --- \| --- \| | 1. Single  2. Married  3. Divorced  4. Widowed  5. Separated  6. Other (specify)--------- | | | | | |  | |  |
| 104 | What is your religion? | 1. Orthodox  2. Muslim  3. Protestant  4. Catholic  5. Other specify_____ | | | | | |  | |  |
| 105 | Ethnicity? | 1. Amhara  2. Oromo  3. Tigrie  4.Guraghe  5.Wolayta  6.. Other (specify ------ | | | | | |  | |  |
| 106 | \| What level of schooling have you completed? \|  \| \| --- \| --- \| | 1. Not read and write  2. Read and write  3. Primary school (Grade-8)  4. Secondary school (Grade 9-10)  5. Preparatory school (Grade 11-12)  6. Certificate  7. Diploma  8. Degree and above | | | | | |  | |  |
| 107 | What is your main occupation? | 1. Housewife  2. Farmer  Government employee  4. Private employee  NGO employee  Daily labourer  7. Student  8. Other (Specify)------------ | | | | | |  | |  |
| 108 | Who is the head of the household? | Housebound 2. Wife | | | | | |  | |  |
| 109 | What is the age of household head | ________ | | | | | |  | |  |
| 110 | What level of education the father has completed? | ________ | | | | | |  | |  |
| 111 | What is your family size? | ________ | | | | | |  | |  |
| 112 | Please specify family size with age composition | _____children less than 2 years’ old  _____children between age 2 and 5  _____children greater than 5 years’ old | | | | | |  | |  |
| **Section two: Household wealth** | | | | | | | | | |  |
| 201 | Who owns the home you are living now? | 1. Private  2. Government  3. Rent  4. Other (specify)_____ | | | | | |  | |  |
| 202 | In your house how many rooms are there? | _____Rooms | | | | | |  | |  |
| 203 | How many rooms in the household are used for sleeping? | ______Rooms | | | | | |  | |  |
| 204 | What are the main walls made of in your house? | 1. Bamboo  2. wood with mud  3. Uncovered adobe  4. Covered adobe  5. Play wood  6. Carton  7. Cement  8. Stone with lime/cement  9. Cement blocks  10. Wood planks  11. Corrugated iron  12. Other (please Specify) ____________ | | | | | |  | |  |
| 205 | What is the main material of the roof in your house? | 1. 1. Corrugated Iron 2. 2. Wood 3. 3. Cement Fibber   4. Roofing Shingles  5. Leaf  6. Plastic Sheets  7. Bamboo  8. Other (please Specify) | | | | | |  | |  |
| 206 | Does the house have separated kitchen for cooking? | 1. 1. Yes 2. 2. No | | | | | |  | |  |
| 207 | What kind of toilet facility does your household have? | 1. Pour flush toilet  2. Ventilated improved pit latrine  3. Pit latrine with slab  4. Pit latrine without a slab  5. No latrine  6. Other (specify)_____ | | | | | |  | |  |
| 208 | What is the main source of drinking water for members of your household? | 1. Piped water  2. Protected well  3. Unprotected well  4. Protected spring  5. Unprotected spring  6. Surface water (River/stream/  7. Pond/lake//Dam)  8. Tanker  9. Bottled water  10. Other (specify)____ | | | | | |  | |  |
| 209 | Does the household own agricultural land? | 1.Yes  2. No | | | | | |  | | If no skip to q211 |
| 210 | How many (local units) of agricultural land do members of this household own? | ______Local units | | | | | |  | |  |
| 211 | How many of the following animals does this household own? | Item | | | Unit | | |  | |  |
|  |  | 1. Cattle | | |  | | |  |  |  |
|  |  | 1. Milk cows | | |  | | |  |  |  |
|  |  | 1. Horses, donkey | | |  | | |  |  |  |
|  |  | 1. Chickens | | |  | | |  |  |  |
|  |  | 1. Goats | | |  | | |  |  |  |
|  |  | 1. Sheep | | |  | | |  |  |  |
|  |  | 1. Others(specify)_ | | |  | | |  |  |  |
| 212 | How much of the following items does your household have? | 1. Radio | | |  | | |  | |  |
|  |  | 1. Computer | | |  | | |  | |  |
|  |  | 1. Television | | |  | | |  | |  |
|  |  | 1. Non-mobile telephone | | |  | | |  | |  |
|  |  | 1. Refrigerator | | |  | | |  | |  |
|  |  | 1. Table | | |  | | |  | |  |
|  |  | 1. Chair | | |  | | |  | |  |
|  |  | 1. Bed with cotton | | |  | | |  | |  |
|  |  | 1. Sponge/spring/mattress | | |  | | |  | |  |
|  |  | 1. Sofa | | |  | | |  | |  |
|  |  | 1. An animal-drawn cart | | |  | | |  | |  |
|  |  | 1. Bicycle | | |  | | |  | |  |
|  |  | 1. Car or truck | | |  | | |  | |  |
|  |  | 1. Bajaj | | |  | | |  | |  |
|  |  | 1. Mobile phone | | |  | | |  | |  |
| 213 | Does a member of this household have a bank/microfinance account? | 1.yes  2.no | | |  | | |  | | If no skip Q214 |
| Household Information about Nutrition Sensitive Agriculture program | | | | | | | | | | |
| 214 | Is your household beneficiary of Nutrition Sensitive Agriculture program | 1.yes  2.no | | |  | | |  | |  |
| 215 | For how money years your household was beneficiary of the program? | ---------- | | |  | | |  | |  |
| **Section Three: Assessment of household food security by HFIAS**  **N.B X-implies times** | | | | | | | | | |  |
| 301 | In the past four weeks, did you worry that your household would not have enough food? | 1.Yes  2.No | | | | | |  | | If no skip Q301a |
| 301a | How often did this happen? | 1 = Rarely (1X or 2X in the past four weeks)  2 = Sometimes (3-10X in the past four weeks)  3 = Often (>10 X in the past 4 weeks) | | | | | |  | |  |
| 302 | In the past four weeks, were you or any household member not able to eat the kinds of foods you preferred because of a lack of resources? | 1.Yes  2.No | | | | | |  | | If no skip Q302a |
| 302a | How often did this happen? | 1 = Rarely (1X or 2X in the past four weeks)  2 = Sometimes (3-10X in the past four weeks)  3 = Often (>10 X in the past 4 weeks) | | | | | |  | |  |
| 303 | In the past four weeks, did you or any household member have to eat a limited variety of foods due to a lack of resources? | 1.Yes  2.No | | | | | |  | | If no skip Q303a |
| 303a | How often did this happen? | 1 = Rarely (1X or 2X in the past four weeks)  2 = Sometimes (3-10X in the past four weeks)  3 = Often (>10 X in the past 4 weeks) | | | | | |  | |  |
| 304 | In the past four weeks, did you or any household member have to eat some foods that you did not want to eat because of a lack of resources to obtain other food? | 1.Yes  2.No | | | | | |  | | If no skip Q304a |
| 304a | How often did this happen? | 1 = Rarely (1X or 2X in the past four weeks)  2 = Sometimes (3-10X in the past four weeks)  3 = Often (>10 X in the past 4 weeks) | | | | | |  | |  |
| 305 | In the past four weeks, did you or any household member have to eat a smaller meal than you felt you needed because there was not enough food? | 1.Yes  2.No | | | | | |  | | If no skip 305a |
| 305a | How often did this happen? | 1 = Rarely (1X or 2X in the past four weeks)  2 = Sometimes (3-10X in the past four weeks)  3 = Often (>10 X in the past 4 weeks) | | | | | |  | |  |
| 306 | In the past four weeks, did you or any other household member have to eat fewer meals in a day because there was not enough food? | 1.Yes  2.No | | | | | |  | | If no skip 306a |
| 306a | How often did this happen? | 1 = Rarely (1X or 2X in the past four weeks)  2 = Sometimes (3-10X in the past four weeks)  3 = Often (>10 X in the past 4 weeks) | | | | | |  | |  |
| 307 | In the past four weeks, was there ever no food to eat of any kind in your household because of lack of resources to get food? | 1.Yes  2.No | | | | | |  | | If no skip 307a |
| 307a | How often did this happen? | 1 = Rarely (1X or 2X in the past four weeks)  2 = Sometimes (3-10X in the past four weeks)  3 = Often (>10 X in the past 4 weeks) | | | | | |  | |  |
| 308 | In the past four weeks, did you or any household member go to sleep at night hungry because there was not enough food? | 1.Yes  2.No | | | | | |  | | If no skip to Q308a |
| 308a | How often did this happen? | 1 = Rarely (1X or 2X in the past four weeks)  2 = Sometimes (3-10X in the past four weeks)  3 = Often (>10 X in the past 4 weeks) | | | | | |  | |  |
| 309 | In the past four weeks, did you or any household member go a whole day and night without eating anything because there was not enough food? | 1.Yes  2.No | | | | | |  | | If no skip to Q309a |
| 309a | How often did this happen? | 1 = Rarely (1X or 2X in the past four weeks)  2 = Sometimes (3-10X in the past four weeks)  3 = Often (>10 X in the past 4 weeks) | | | | | |  | |  |
| 310 | When there is shortage of food in the household what copping strategies do you and your family apply? | 1. Reduce the amount of food we eat  2. Reduce the number of times? Frequency we eat per day  3. By changing the quality and price of reduced foods  4. Increasing our income by doing more work  5. Reducing the cost of non-food items  6. Selling furniture and property  7. Receiving food or financial assistance  8. By borrowing from a bank or other loan  9. Mention any other……… | | | | | |  | |  |
| **Section four: Assessment child dietary diversity score**  The next question focuses the seven-child dietary diversity or varieties of foods from the lists of food with one type or conjugates within 24 hours from yesterday 12:00 to today 12:00 o’clock during the day or night, did the child drink/eat any food items mentioned below? Encircle yes (1) if the child consumed, or encircle No (2) if the child not consumed. | | | | | | | | | | |
| \| No \|  \|  \|  \| \| --- \| --- \| --- \| --- \| | Questions and filters | | | | Yes (1) | | No (2) | Remark | | |
| 401 | Porridge, bread, rice, noodles, or other foods made from grains | | | | 1 | | 2 |  | | |
| 402 | Pumpkin, carrots, squash, or sweet potatoes that are yellow or orange inside | | | | 1 | | 2 |  | | |
| 403 | White potatoes, white yams, manioc, cassava, or any other foods made from  roots | | | | 1 | | 2 |  | | |
| 404 | Any dark green leafy vegetables | | | | 1 | | 2 |  | | |
| 405 | other vegetables (e.g. tomato, onion, eggplant), including wild vegetables | | | | 1 | | 2 |  | | |
| 406 | Ripe mangoes, cantaloupe, apricots (fresh or dried), ripe papaya, dried peaches + another locally available vitamin A-rich fruits | | | | 1 | | 2 |  | | |
| 407 | Any other fruits or vegetables | | | | 1 | | 2 |  | | |
| 408 | liver, kidney, heart, or other organ meats | | | | 1 | | 2 |  | | |
| 409 | any meat, such as beef, pork, lamb, goat, chicken, or duck | | | | 1 | | 2 |  | | |
| 410 | Egg (chicken, duck, guinea fowl or any other egg) | | | | 1 | | 2 |  | | |
| 411 | Fish (fresh or dried fish or shellfish) | | | | 1 | | 2 |  | | |
| 412 | beans, peas, lentils, nuts, seeds or foods made from these | | | | 1 | | 2 |  | | |
| 413 | Milk (including breast milk), cheese, yoghurt or other milk products except Breast milk | | | | 1 | | 2 |  | | |
| 414 | oil, fats or butter added to food | | | | 1 | | 2 |  | | |
| 415 | sugar, honey, sweetened soda, sweetened juice or sugary foods such as chocolates, candies, cookies | | | | 1 | | 2 |  | | |
| 416 | spices (black pepper, salt), condiments (soy sauce, hot sauce), coffee, tea, alcoholic beverages. | | | | 1 | | 2 |  | | |
| **Section Five: Vaccination, wellbeing and Nutritional Status assessments of children’s aged 6 months-5 years** | | | | | | | | | | |
| 501 | What is the sex of the child? | 1.Male  2.Female | | | | | |  |  | |
| 502 | What is the age of the child? | ______ | | | | | |  |  | |
| 503 | What is his/her birth order? | ______th | | | | | |  |  | |
| 504 | Have you told by a health professional about the child has experienced any illness in the last 2 weeks? | 1.Yes  2.No | | | | | |  | If no skip to Q506 | |
| 505 | If yes to Q 504, what type of illness did you experienced? | 1. diarrhoea  2. Malaria  3. AURTI  4. Parasitic infection  5. Others (specify)_____________ | | | | | |  |  | |
| 506 | Does the child have started complementary feeding? | 1.Yes  2.No | | | | | |  | If no skip to Q 508 | |
| 507 | At what age, complementary feeding was started? | --------------- | | | | | |  |  | |
| 508 | Does the child have a habit of washing hand before going to meal? | 1. Yes  2. No 3. Does not apply to the child | | | | | |  |  | |
| 509 | Current weight (kg) (Record repeated measurements) | 1^st^ measurement | 2^nd^ measurement | | | 3^rd^ measurement | Average |  |  | |
|  |  | --------Kg | ------Kg | | | -----Kg | --------Kg |  |  |  |
| 510 | Height/length | --------Cm | ------Cm | | | ----Cm | -------Cm |  |  | |
| 511 | MUAC | --------Cm | -----Cm | | | ----Cm | ------Cm |  |  | |
| 512 | Does the child have received the following vaccinations? Please tick on the corresponding box below, check the child immunization card. | | | | | | | | | |
|  |  | Yes | | No | | | |  |  | |
|  | BCG |  | |  | | | |  |  | |
|  | Oral Polio Vaccine (OPV) 0 |  | |  | | | |  |  | |
|  | Oral Polio Vaccine (OPV) 1 |  | |  | | | |  |  | |
|  | Oral Polio Vaccine (OPV) 2 |  | |  | | | |  |  | |
|  | Oral Polio Vaccine (OPV) 3 |  | |  | | | |  |  | |
|  | Pentavalent 1 |  | |  | | | |  |  | |
|  | Pentavalent 2 |  | |  | | | |  |  | |
|  | Pentavalent 3 |  | |  | | | |  |  | |
|  | Pneumococcal 1 |  | |  | | | |  |  | |
|  | Pneumococcal 2 |  | |  | | | |  |  | |
|  | Pneumococcal 3 |  | |  | | | |  |  | |
|  | Rotavirus 1 |  | |  | | | |  |  | |
|  | Rotavirus 2 |  | |  | | | |  |  | |
|  | Measles |  | |  | | | |  |  | |
|  | Vitamin A (Most Recent) |  | |  | | | |  |  | |
| **Part six: Assessment of maternal previous history and current Nutrition Status** | | | | | | | | | | |
| 601 | What was your pre-pregnancy weight (of the last pregnancy)? | 1. In kilogram______  I don’t remember | | | | | |  |  | |
| 602 | Mother received ANC follow-up  for the child being interviewed. | 1.Yes  2.No 3. I don’t remember | | | | | |  | If no skip to Q604 | |
| 603 | What is the total number of ANC follow-up the mother received? |  | | | | | |  |  | |
| 604 | Mother received PNC follow-up for the child being interviewed. | 1.Yes  2. No 3.I don’t remember | | | | | |  |  | |
| 605 | What is the total number of PNC follow-up the mother received? |  | | | | | |  |  | |
| 606 | During pregnancy or lactation, did you consume extra food? (for the child included in the study) |  | | | | | |  |  | |
| 607 | Have you ever used family planning methods? | 1. Yes, if yes please specify……. 2. No | | | | | |  |  | |
| 608 | If you say yes to question number 607, what type of contraceptive was you using? |  | | | | | |  |  | |
| 608 | For how long the child exclusively breast feed? |  | | | | | |  |  | |
| 609 | For how long do you think should a child exclusively be breast-fed? | 1……month | | | | | |  |  | |
| 610 | Current maternal Height (Record repeated measurements) | 1^st^ measurement | 2^nd^ measurement | | | | Average |  |  | |
|  |  | ………...cm | …………...cm | | | | ……...cm |  |  |  |

**Thank you for completing the questionnaire!**

**Anthropometric Measurement Protocols and Procedures**

**Child Height and Length**

1. Standing height was measured with a fixed studio-meter with a vertical backboard and a moveable headboard. Recumbent length was used for children under 2 years of age and measured using an infant-meter with a fixed head piece and horizontal backboard, and an adjustable foot piece.
2. Identify the subject and explain the procedure.
3. Have the child remove hair ornaments, ponytails, buns, braids, etc.
4. Ask/asist the child to remove shoes except for diapers or underpants.
5. For children age greater than 2 years have the child stand on the floor with the heels of both feet together and the toes pointed slightly outward. Body weight is evenly distributed and both feet are flat on the floor.
6. Align the childs head in the Frankfort horizontal plane (the horizontal line from the ear canal to the lower border of the orbit of the eye is parallel to the floor and perpendicular to the vertical backboard.
7. Arms should hang free at the sides with palms facing the thighs.
8. Measurer's eyes should be level with the headboard.
9. Position the headboard firmly on top of the head with enough pressure to compress the hair.
10. For the child less than 2 years, lay the child on a measuring board on the infant-meter with the feet toward the foot piece and the head against the fixed head piece. The parent checks the position of the heels, buttocks, shoulder blades, and the back of the head for contact with the vertical backboard the second anthropometrist record the data.
11. The measurement is recorded to the nearest 0.1 cm.

**Weight**

Children were weighed in kilograms using a digital calibrated weight scale (salter scale). Children wear only diapers. Infants was weighed with the assistance of an adult. The parent, guardian, or the examiner stand alone on the scale while the recorder shows the weight and the other recorder captures the result to the nearest 0.1 kg. The child is then handed to the parent or caregiver on the scale.

**Amharic Version Questionnaire**

|  | ቃለ መጠይቁ የትካሄደበት ቀን_______________________  የጠያቂዉ ስም_________________________________________  ለሚቀጥለዉ የጉበኝት ቀን ቀጠሮ_______________________  የተጠያቂዉ መለያ ቁጥር________________________________ | | | | | | | |  | |  |
| --- | --- | --- | --- | --- | --- | --- | --- | --- | --- | --- | --- |
| ተ.ቁ | መጠይቅ | መልስ | | | | | ኮድ | | ዝለል | |  |
| **ክፍል 1. አጠቃላይ ማህበራዊ-ኢኮኖሚያዊ መለያ መጠይቅ** | | | | | | | | | | | |
| 101. | የእናት እድሜ ስንት ነዉ? | _______________________ | | | | |  | |  | |  |
| 102. | የመኖሪያ ቦታዎ የት ነዉ? | 1. ከተማ  2 ገጠር | | | | |  | |  | |  |
| 103. | የእናት የትዳር ሁኔታ ምንድነዉ? | 1.ያላገባች  2. ያገባች  3. የፈታች  4.ባል የሞተባት  5. ተለያይተዉ የሚኖሩ  6.ሌላ_______________ | | | | |  | |  | |  |
| 104 | የሚከተሉት የሃይማኖት እምነት ምንድን ነዉ? | 1. ኦርቶዶክስ ክርስትያን  2. ሙስሊም  3. ፕሮትስታንት  4. ካቶሊክ  5. ሌላ___________________ | | | | |  | |  | |  |
| 105 | ስታገቢ ዕድሜሽ ስንት ነበር? | ________________________ | | | | |  | |  | |  |
| 106 | ብሄርሽ ምንድን ነዉ? | 1.አማራ  2.ኦሮሞ  3.ትግሬ  4.ጉራጌ  5.ሌላ________________ | | | | |  | |  | |  |
| 107 | የትምህርት ደረጃሽ ስንት ነዉ? | 1.ማንበብ እና መፃፍ የማትችል  2. ማነበብና መፃፍ የምትችል  3. አንደኛ ደረጃ (1-8)  4.ሁለተኛ ደረጃ (9-10)  5.መሰናዶ  6.ሰርተፍኬት  7. ዲፕሎማ  8. ዲግሪ  9.ማሰትር እና ከዛ በላይ | | | | |  | |  | |  |
| 108 | ዋና ስራሽ መንድነዉ? | 1.የቤት አመቤት  2.ገበሬ  3.የመንግስት ተከፋይ  4.የግል መስሪያ ቤት ተከፋይ  5.የልማት አጋር ድርጅት ተከፋይ  6.የቀን ሰራተኛ  7.ተማሪ  8.ሌላ………. | | | | |  | |  | |  |
| 109 | የቤቱ ሃላፊ ማን ነዉ? | 1.ባል  2.ሚስት | | | | |  | |  | |  |
| 110 | የባለቤትሽ ዕድሜ ስንት ነዉ? | …………….. | | | | |  | |  | |  |
| 111 | የባለቤትሽ የትምህርት ደረጃ ስንት ነዉ? | 1.ማንበብ እና መፃፍ የማትችል  2. ማነበብና መፃፍ የምትችል  3. አንደኛ ደረጃ (1-8)  4.ሁለተኛ ደረጃ (9-10)  5.መሰናዶ  6.ሰርተፍኬት  7. ዲፕሎማ  8. ዲግሪ  9.ማሰትርስ እና ከዛ በላይ | | | | |  | |  | |  |
| 112 | አጠቃላይ የቤተሰብ ብዛት ስንት ነው? | ------------ | | | | |  | |  | |  |
| 113 | አባክዎ የልጆችዎትን የዕድሜ ደረጃ ስንተ እንደሆን በዝረዝር ይንገሩኝ? | 1.ከ2 አመት በታች_______________  2.ከ2 እስከ 5 አመት___________  3.ከ5 አመት በላይ_____________ | | | | |  | |  | |  |
|  | **ክፍል 2. የቤተሰብ ምጣኔ ሁኔታ ለመዳሰስ የተዘጋጅ መጠይቅ** | | | | | | | | | |  |
| 201 | የምትኖሩበት ቤት የማን ነዉ? | 1.የራሳችን (የግል)  2.የመንግስት ኪራይ  3.የግል ኪራይ  4.ሌላ | | | | |  | |  | |  |
| 202 | የመኖሪያ ቤታችሁ ስነት ክፍሎች አሉት? | ________________ | | | | |  | |  | |  |
| 203 | ለመኝታ ክፍል የተለየ ስንት ክፍሎች አሉት? | ­­­­­­________________ | | | | |  | |  | |  |
| 204 | የቤቱ ግድግዳ በዋነኝነት የተሰራዉ ከምንድን ነዉ? | 1.ከሸምበቆ/ቀርቀሃ  2.ከእንጨትና ከጭቃ  3.ከጡብ  4.ከካረቶን  5.ከሲሚንቶ  6.ከድንጋይ እና ሲሚንቶ  7.ከብሎኬት  8.የእንጨየት ጨፈቃ  9.ቆርቆሮ  10.ሌላ________________________ | | | | |  | |  | |  |
| 205 | የቤቱ ጣሪያ በዋነኝነት የተሰራዉ ከምንድን ነዉ? | 1.ከቆርቆሮ  2.ከእንጨት  3.ከሲሚንቶ  4.ከሸክላ ቆርቆሮ  5.ሳር/ቅጠል  6.ከፕላሰቲክ/ሸራ/ጨርቅ  7.ቀርቀሃ  8. ሌላ | | | | |  | |  | |  |
| 206 | ቤቱ ራሱን የቻል የተለየ ኩሽና ቤት አለዉ? | 1.አዎ  2.የለም | | | | |  | |  | |  |
| 207 | ምን አይነት መፀዳጃ ቤት ነዉ አብዛኛዉ የቤተሰብ አባል የሚጠቀመዉ? | 1.በዉሃ የሚሰራ መፀዳጃ ቤት  2.የተሻሻለ መፀዳጃ ቤት  3.የተለመደ መፀዳጃ ቤት/ክዳን ያለዉ  4.የተለመደ መፀዳጃ ቤት/ክዳን የሌለዉ  5. ጫካ/ቁጥቃጦ  6.ሌላ_____________ | | | | |  | |  | |  |
| 208 | የቤተሰብ አባላት የመጠጥ ዉሃ የሚያገኙት ከየት ነዉ? | 1.ከቧንቧ  2.ከታጠረ ጉዳጓድ  3. ካልታጠረ ጉዳጓድ  4.ከታጠረ ምንጭ  5. ካልታጠረ ምንጭ  6. ወራጅ ዉሃ/የወንዝ  7. ኩሬ/ግድብ  8. ሮቶ/ታነከር  9.የታሸገ ሃይላንድ ዉሃ  10.ሌላ_____________________ | | | | |  | |  | |  |
| 209 | በቤተሰብ አባላት ስም የተመዘገበ የእረሻ መሬት አላችሁ? | 1.አዎ  2.የለም | | | | |  | |  | |  |
| 210 | ለጥያቄ ቁጥር 122 አዎ ከሆነ መልስዎ ስንት ሄከታር የእርሻ መሬት አላችሁ (በአካባቢዉ መለኪያ ይጠይቁ)? | _____________________ | | | | |  | |  | |  |
| 211 | ከሚከተሉት የቤት እንስሳት ቤተሰቡ ምን ያህል አለዉ? | አይነት | | **(1)** አዎ ብዛት***(*√)** | **(2)**የለም**(√)** | |  | |  | |  |
|  |  | ሀ. የቀንድ ከብት (በሬ፤ወይፈን) | |  |  | |  |  |  |  |  |
|  |  | ለ. የወተት ላም | |  |  | |  |  |  |  |  |
|  |  | ሐ. ሌላ የቀንድ ከብት (ጥጃ፤ ጊደር) | |  |  | |  |  |  |  |  |
|  |  | መ. ፈረስ፤በቀሎ፤አህያ | |  |  | |  |  |  |  |  |
|  |  | ሠ. ዶሮ | |  |  | |  |  |  |  |  |
|  |  | ረ. ፍየል | |  |  |  |  |  |  |  |  |
|  |  | ሰ. በግ | |  |  |  |  |  |  |  |  |
|  |  | ሸ.ሌላ | |  |  |  |  |  |  |  |  |
| 212 | ቤተሰቡ ከሚከተሉት ዉስጥ ምን ምን አለዉ? | የእቃ አይነት | | **(1)** አዎ ***(*√)** | **(2)**የለም **(√)** | |  | |  | |  |
|  |  | ሀ. ሬዲዮ | |  |  | |  |  |  |  |  |
|  |  | ለ. ኮምፒዉተር | |  |  | |  |  |  |  |  |
|  |  | ሐ. ቴሌቪዥን | |  |  | |  |  |  |  |  |
|  |  | መ. የቤት ስልክ | |  |  | |  |  |  |  |  |
|  |  | ሠ.ፍሪጅ | |  | |  |  |  |  |  |  |
|  |  | ረ. ጠረምፔዛ | |  | |  |  |  |  |  |  |
|  |  | ሰ.ወንበር | |  | |  |  |  |  |  |  |
|  |  | ሸ. አልጋ | |  | |  |  |  |  |  |  |
|  |  | ቀ.ፍራሽ | |  | |  |  |  |  |  |  |
|  |  | በ. ሶፋ | |  | |  |  |  |  |  |  |
|  |  | ተ. እንስሳት የሚነቀሳቀስ ጋሪ | |  | |  |  |  |  |  |  |
|  |  | ቸ. ሳይክል | |  | |  |  |  |  |  |  |
|  |  | ኀ. ሞተር ሳይክል | |  | |  |  |  |  |  |  |
|  |  | ነ. መኪና/ትረክ | |  | |  |  |  |  |  |  |
|  |  | ኘ. ባጃጅ | |  | |  |  |  |  |  |  |
|  |  | አ. ተንቀሳቃሽ ሞባይል | |  | |  |  |  |  |  |  |
| 213 | የቤተሰብ አባል ዉስጥ የባንክ/ብድር እና ቁጠባ ደብተር ያለዉ ስዉ አለ? | 1.አዎ  2.ለም | | | | |  | |  | |  |
|  | **ስለ ቤተሰቡ የግብርና ማሻሻያ ፐሮግራሞች ተጠቃሚነት መረጃ** | | | | | | | |  | |  |
| 214 | የእርስዎ ቤተሰብ የተመጣጠነ ምግብን ተደራሽ ለማደረግ የሚተገበር ፕሮግራም ተጠቃሚ ነዎት? | 1.አዎ  2.ለም | | | | |  | |  | |  |
| 215 | አዎ ካሉ ለስንት አመት ተጠቃሚ ነበሩ? | ---------- | | | | |  | |  | |  |
|  | ክፍል 3. የቤተሰብ የምግብ ዋስትና ሁኔታ ለመዳሰስ የተዘጋጀ መጠይቅ | | | | | | | | | |  |
| 301 | ባለፈው አንድ ወር ውስጥ በቤትዎ ውስጥ የምግብ እጥረት እዳያጋጥሞት ተጨንቀው ያውቃሉ? | 1. አዎ  2.አላውቅም (የለም ካሉ ጥያቄ 301a ይዝለሉት) | | | | |  | |  | |  |
| 301a | መልስዎ አዎ ከሆነ ይህ ለምን ያህል ግዜ ተከስቷል? | 1.አልፎ አልፎ (አንዴ ወይ ሁለቴ)  2. የተወሰነ ግዜ (3-10)  3.ብዙ ግዝ (ከ10 ግዜ በላይ) | | | | |  | |  | |  |
| 302 | ባለፍው እንድ ወር ውስጥ እርሰዎ ወይም ሌላ የቤተሰብ አባል በምግብ እጥረት ምክኒያት የሚፈልጉትን ምግብ ሳይመገቡ ቀርተዋል? | 1.አዎ  2.አላውቅም (የለም ካሉ ጥያቄ 302a ይዝለሉት) | | | | |  | |  | |  |
| 302a | መልስዎ አዎ ከሆነ ይህ ለምን ያህል ግዜ ተከስቷል? | 1.አልፎ አልፎ (አንዴ ወይ ሁለቴ)  2.የተወሰነ ጊዜ (3-10)  3.ብዙ ጊዜ (ከ10 ግዜ በላይ) | | | | |  | |  | |  |
| 303 | ባለፈው አንድ ወር ወስጥ እርሶ ወይም ሌላ የቤተሰብ አባል በምግብ አቅርቦት ምክኒያት የሚመገቧቸው ምግብ አይነቶች ቀንሰዋል? | 1.አዎ  2.አላውቅም (የለም ካሉ ጥያቄ 303a ይዝለሉት) | | | | |  | |  | |  |
| 303a | መልስዎ አዎ ከሆነ ይህ ለምን ያህል ጊዜ ተከስቷል? | 1.አልፎ አልፎ (አንዴ ወይ ሁለቴ)  2.የተወሰነ ግዜ (3-10)  3.ብዙ ግዝ (ከ10 ግዜ በላይ) | | | | |  | |  | |  |
| 304 | ባለፈው አንድ ወር ውስጥ እርሶ ወይም ሌላ የቤተሰብ አባል በምግብ አቅርቦት ምክኒያት የማይፈልጉትን የምግብ አይነት ተመግበዋል? | 1.አዎ  2.አላውቅም (የለም ካሉ ጥያቄ 304a ይዝለሉት | | | | |  | |  | |  |
| 304a | መለስዎ አዎ ከሆነ ይህ ለምን ያህል ጊዜ ተከስቷል? | 1.አልፎ አልፎ (አንዴ ወይ ሁለቴ)  2.የተወሰነ ግዜ (3-10)  3.ብዙ ግዝ (ከ10 ግዜ በላይ) | | | | |  | |  | |  |
| 305 | ለፈው አንድ በወር ውስጥ እርስዎ ወይም ሌላ ቤተሰብ አባል በምግብ እጥረት ምክኒያት የሚመገቡትን የምግብ መጠን ቀንሰዋል? | 1.አዎ  2.አላውቅም (የለም ካሉ ጥያቄ 305a ይዝለሉት) | | | | |  | |  | |  |
| 305a | መልሶ አዎ ከሆነ ይህ ለምንህል ጊዜ ተከስቷል? | 1.አልፎ አልፎ (አንዴ ወይ ሁለቴ)  2.የተወሰነ ግዜ (3-10)  3.ብዙ ግዝ (ከ10 ግዜ በላይ) | | | | |  | |  | |  |
| 306 | ባለፈው አንድ ወር ውስጥ በምግብ እጥረት ምክኒያት እርሶ ወይም ሌላ የቤተሰብ አባል በምግብ እጥረት ምክኒያት በቀን ምግብ የሚበሉባችው ግዜያቶች ቀንሰዋል? | 1.አዎ  2.አላውቅም (የለም ካሉ ጥያቄ 306a ይዝለሉት) | | | | |  | |  | |  |
| 306a | መልሱ አዎ ከሆነ ይህ ለምንህል ጊዜ ተከስቷል? | 1.አልፎ አልፎ (አንዴ ወይ ሁለቴ)  2.የተወሰነ ግዜ (3-10)  3.ብዙ ግዝ (ከ10 ግዜ በላይ) | | | | |  | |  | |  |
| 307 | ባለፈው አንድ ወር ውስጥ በምግብ እጥረት ምክኒያት ማንኛውም የሚበላ ምግብ ከቤት ጠፍቶ ያውቃል? | 1.አዎ  2.አላውቅም (የለም ካሉ ጥያቄ 307 a ይዝለሉት) | | | | |  | |  | |  |
| 307a | መልሶ አዎ ከሆነ ይህ ለምንህል ጊዜ ተከስቷል? | 1.አልፎ አልፎ (አንዴ ወይ ሁለቴ)  2.የተወሰነ ግዜ (3-10)  3.ብዙ ግዝ (ከ10 ግዜ በላይ) | | | | |  | |  | |  |
| 308 | ባለፈው አንድ ወር ውስጥ እርሶ ወይም ሌላ የቤተሰብ አባል በምግብ እጥረት ምክኒያት እየተራቡ ምግብ ሳይበሉ ተኝተው ያውቃሉ? | 1.አዎ  2.አላውቅም (የለም ካሉ ጥያቄ 308 a ይዝለሉት) | | | | |  | |  | |  |
| 308a | መልሱ አዎ ከሆነ ይህ ለምንህል ጊዜ ተከስቷል? | 1.አልፎ አልፎ (አንዴ ወይ ሁለቴ)  2.የተወሰነ ግዜ (3-10)  3.ብዙ ግዝ (ከ10 ግዜ በላይ) | | | | |  | |  | |  |
| 309 | ባለፈው አንድ ወር ውስጥ ማንኛውም የቤተሰብ አባል በምግብ እጥረት ምክኒያት እየተራቡ ቀንና ለሊት ሙሉ ምግብ ሳይበሉ ቀርተው ያውቃሉ? | 1.አዎ  2.አላውቅም (የለም ካሉ ጥያቄ 309a ይዝለሉት) | | | | |  | |  | |  |
| 309a | መልሶ አዎ ከሆነ ይህ ለምንህል ጊዜ ተከስቷል? | 1.አልፎ አልፎ (አንዴ ወይ ሁለቴ)  2.የተወሰነ ግዜ (3-10)  3.ብዙ ግዝ (ከ10 ግዜ በላይ) | | | | |  | |  | |  |
| 310 | እርሶና ቤተሰቦዎ የምግብ እጥረት በሚያጋጥሞት ጊዜ ምን አይነት መቋቋሚያ ስልቶች ተጠቅመው ያውቃሉ? ((ከአንድ በላይ መልስ መስጠት ይቻላል) | 1.የምመገበውን የምግብ መጠን መቀነስ  2.በቀን የሚመገቡበት ጊዜ መቀነስ  3.ጥራታቸውንና ዋጋቸው የቀነሱ ምግቦች በመቀየር  4.ተጨማሪ ስራ በመስራት ገቢያችን በመጨመር  5.ምግብ ነክ ያልሆኑ ነገሮችን ወጪ በመቀነስ  6.የቤት እቃዎችንና ንብረቶችን በመሸጥ  7.የምግብ ወይም የገንዘብ እርዳታ በመቀበል  8.ከባንክ ወይም ከሌላ ብድር በመውሰድ  9.ሌላ ካለ ይጥቀሱ--------------- | | | | |  | |  | |  |
|  | **ክፍል 4. የህፃናትን የተለያዩ የምግብ አይነቶችና የአመጋገብ ስሌት ለመዳሰስ የተዘጋጀ መጠይቅ**  አሁን 24 ሰአት ውስጥ ህፃንዎ የተለያዩ አይነት ምግቦች ተመግበው እንደሆን እጠይቀዎትአለሁ:: እርሰዎም በእያንዳንዱ የምግብ ምድቦች ተመግቡል ወይም አልትመገበም በማለት ይመልሳሉ:: | | | | | | | | | |  |
|  |  | | **(1)** አዎ ***(*√)** | | **(2)** የለም **(√)** | | | | | **ኮድ** |  |
| 401 | ገንፎ ፣ ዳቦ ፣ ሩዝ ፣ ኑድል ወይም ከእህል የሚዘጋጁ ሌሎች ምግቦች(ጤፍ ስንዴ ገብስ በቀሎ ማሩዝ እንዲሁም የነዚህ ውጤት የሆኑት ገንፎ ፓስታ ማካሮኒና ሌሎችም | | 1 | | 2 | | | | |  |  |
| 402 | ዱባ ፣ ካሮት ፣ ዱባ ፣ ወይም ቢ ውስጥ ወይም ብርቱካናማ የሆኑ ጣፋጭ ድንች | | 1 | | 2 | | | | |  |  |
| 403 | ነጭ ድንች ፣ ነጭ ያም ፣ ማኒዮክ ፣ ካሳቫ ፣ ወይም ከማንኛውም ሌላ ሥሮች የተሰሩ ምግቦች | | 1 | | 2 | | | | |  |  |
| 404 | ማንኛውም ጥቁር አረንጓዴ ቅጠል ያላቸው አትክልቶች | | 1 | | 2 | | | | |  |  |
| 405 | ሌሎች አትክልቶች | | 1 | | 2 | | | | |  |  |
| 406 | የበሰለ ማንጎ ፣ የበሰለ ፓፓያዎች ፣ ወይም (ሌሎች የአከባቢ ቫይታሚን ኤ የበለፀጉ ፍራፍሬዎች | | 1 | | 2 | | | | |  |  |
| 407 | ሌሎች ፍራፈሬዎች | | 1 | | 2 | | | | |  |  |
| 408 | ጉበት ፣ ኩላሊት ፣ ልብ ወይም ሌላ የአካል ክፍሎች ሥጋ | | 1 | | 2 | | | | |  |  |
| 409 | ማንኛውም ሥጋ ፣ ለምሳሌ የበሬ ፣ የአሳማ ሥጋ ፣ የበግ ፣ የፍየል ፣ የዶሮ ወይም የዳክዬ | | 1 | | 2 | | | | |  |  |
| 410 | እንቁላል | | 1 | | 2 | | | | |  |  |
| 411 | አሳና የባህር ምግቦች | | 1 | | 2 | | | | |  |  |
| 412 | የቅባት እህል/ባቄላ መሰል ጥራጥሬ | | 1 | | 2 | | | | |  |  |
| 413 | ወተትና የወተት ውጤቶች አይብ ፣ እርጎ ወይም ሌሎች የወተት ተዋጽኦዎች | | 1 | | 2 | | | | |  |  |
| 414 | ዘይት/ጮማ | | 1 | | 2 | | | | |  |  |
| 415 | ቾኮሌቶች ፣ ጣፋጮች ፣ ከረሜላዎች ፣ ኬኮች ፣ ኬኮች ፣ ወይም የመሳሰሉት ማንኛውም ጣፋጭ ምግቦች ብስኩት | | 1 | | 2 | | | | |  |  |
| 416 | የተለያየ አይት (ሌሎች ምግቦች) ቅመማ ቅመሞች ፣ የመጠጥ ቅመሞች (ጥቁር በርበሬ ፣ ጨው) ፣ ቅመማ ቅመሞች (አኩሪ አተር ፣ ሞቅ ያለ ድስ) ፣ ቡና ፣ ሻይ ፣ አልኮሆል መጠጦች | | 1 | | 2 | | | | |  |  |
|  | **ክፍል 5. የህፃናትን መለያ፤የክትባት፤ የምግብ እና የጤንነት ሁኔታ ለመዳሰስ እንዲሁም ቁመትና ክብደት ለመለካት የተዘጋጀ ቃለ መጠይቅ** | | | | | | | | | |  |
| 501 | የህፃኑ ፆታ? | | 1.ወንድ  2.ሴት | |  | | | | |  |  |
| 502 | የህፃኑ እድሜ (በወር)? | | ___________________ | |  | | | | |  |  |
| 503 | ህፃኑ ስነተኛ ልጅሽ ነዉ | | **___________________** | |  | | | | |  |  |
| 504 | ህፃኑ ባለፈዉ 2 ሳምንታት ዉስጥ ማንኛዉም አይነት ህመም ታሞ ነበር? | | 1.አዎ  2.የለም (የለም ካሉ ወደ ጥያቄ 505 ይዝለሉ) | |  | | | | |  |  |
| 505 | መልሰዎ አዎ ከሆነ ያመመዉ ምን ነበር? | | 1.ተቅመጥ  2.ወባ  3.የመተነፈሻ አካል ህመም  4.የትላትል በሽታ  5.ሌላ | |  | | | | |  |  |
| 506 | ህፃኑ ተጨማሪ ምግብ ጀምሩል? | | 1.አዎ  2.የለም | |  | | | | |  |  |
| 507 | በስንት ወሩ ነበር ተጨማሪ ምግብ የጀመረዉ? | | **______** | |  | | | | |  |  |
| 508 | ህፃኑ ምግብ ከመመገቡ በፊት እጁን የመተጠብ ልምድ አለዉ? | | 1.አዎ  2.የለም  3.አይመለከተዉም | |  | | | | |  |  |
| 509 | አሁን ያለዉ የህፃኑ ክብደት (በ ኪግ)? | | መጀመሪያ ይለኩ | | ሁለተኛ ይለኩ | | | አማካይ | |  |  |
|  |  |  | **----------------** | | **----------------** | | | **---------** | |  |  |
| 510 | አሁን ያለዉ የህፃኑ ቁመት (በሴ.ሜ)? | | **__________** | | **----------------** | | | **--------** | |  |  |
| 511 | የህፃኑ ክንድ ልኬት (በ ሴ.ሜ)? | | **__________** | | **-----------------** | | | **--------** | |  |  |
|  | **ህፃኑ የሚከተሉትን ክትባቶች ወስዱል፡፡ ከዎሰደ አዎ ካልዎሰደ የለም በማለት ይሙሉ፡፡ እባክዎ የክትባት ካርዱን በማየት ያረጋግጡ** | | | | | | | | | |  |
|  |  | | **(1)** አዎ ***(*√)** | | **(2)** የለም **(√)** | | | | | ኮድ |  |
| 512 | ቢሲጅ | |  | |  | | | | |  |  |
| 513 | ፖሊዮ (OPV)0 | |  | |  | | | | |  |  |
| 514 | ፖሊዮ (OPV)1 | |  | |  | | | | |  |  |
| 515 | ፖሊዮ (OPV)2 | |  | |  | | | | |  |  |
| 516 | ፖሊዮ (OPV)3 | |  | |  | | | | |  |  |
| 517 | አይፒቪ4 | |  | |  | | | | |  |  |
| 518 | ፔንታቫለንት 1 | |  | |  | | | | |  |  |
| 519 | ፔንታቫለንት 2 | |  | |  | | | | |  |  |
| 520 | ፔንታቫለንት 3 | |  | |  | | | | |  |  |
| 521 | ፒሲቪ 1 | |  | |  | | | | |  |  |
| 522 | ፒሲቪ 2 | |  | |  | | | | |  |  |
| 523 | ፒሲቪ 3 | |  | |  | | | | |  |  |
| 524 | ሮታ 1 | |  | |  | | | | |  |  |
| 525 | ሮታ 2 | |  | |  | | | | |  |  |
| 526 | ኩፍኝ 1 | |  | |  | | | | |  |  |
| 527 | ኩፍኝ 2 | |  | |  | | | | |  |  |
|  | **ክፍል 6. የእናቶች የምግብ ሁኔታ ለመዳሰስ እንዲሁም ቁመትና ክብደት ለመለካት የተዘጋጀ ቃለ መጠይቅ** | | | | | | | | | |  |
| 601 | ከእርግዝና በፊት የነበረሽ ክብደት ስንት ነበር? | | **___________** | |  | | | | |  |  |
| 602 | አሁን የሚጠየቀዉን ልጅ እርጉዝ እያለሽ የቅድመ ዎሊድ ክትትል ታደርጊ ነበር? | | 1.አዎ  2.የለም | |  | | | | |  |  |
| 603 | ባጠቃላይ ስንት ጊዜ የቅድመ ዎሊድ ክትትል አድርገሻል | | **__________** | |  | | | | |  |  |
| 604 | አሁን የሚጠየቀዉን ልጅ ከወለድሽ በሁላ የድህረ ዎሊድ ክትትል ታደርጊ ነበር? | | 1.አዎ  2. የለም | |  | | | | |  |  |
| 605 | ባጠቃላይ ስንት ጊዜ የድህር ዎሊድ ክትትል አድርገሻል? | | **________** | |  | | | | |  |  |
| 606 | እረጉዝ እያለሽ የአመጋገብሽ ሁኔታ እንዴት ነበር? | | 1.እንደድሮዉ  2.የጨመረ  3.የቀነሰ  4.አላዉቅም | |  | | | | |  |  |
| 607 | የቤተሰብ ምጣኔ አገልግሎት ተጠቅመሽ ታዉቂያለሽ? | | 1.አዎ  2.የለም | |  | | | | |  |  |
| 608 | መልስሽ አዎ ከሆን የትኛዉን አይነት የወሊድ መከላከያ ነበር በቅርቡ የተጠቀምሽዉ? | | 1.ክኒን  2.መረፌ  3.በክንድ የሚቀበር  4.በማህፀን ዉስጥ የሚቀበር  5. ሌላ_______ | |  | | | | |  |  |
| 609 | ህፃኑን ለስንት ወር ጡት ብቻ አጠባሽዉ? | | **_______________** | |  | | | | |  |  |
| 610 | ህፃንት የእናት ጡት ብቻ እስከ ስንት ወር ድረስ መጥባት አለባቸዉ ብለሽ ታስቢያለሽ? | | **_________________** | |  | | | | |  |  |
| 611 | አሁን ያለዉ የእናት ክብደት (በ ኪ.ግ)? | | **መጀመሪያ ይለኩ** | | **ሁለተኛ ይለኩ** | | **አማካይ** | | |  |  |
|  |  |  | **--------------** | | **-------------** | | **----------** | | |  |  |
| 612 | አሁን ያለዉ የእናት ቁመት (በ ሜትር)? | | **_________________** | | **-------------** | | **------------** | | |  |  |
