# Supplementary material for: Undernutrition and associated factors among children aged 6–59 months in nutrition-sensitive agriculture intervention implemented Basona district, North Shewa Zone, Amhara region, Ethiopia
Source: PLoS One. 2023 Apr 26;18(4):e0284682. doi: 10.1371/journal.pone.0284682 (PMC10132697; doi:10.1371/journal.pone.0284682)
Supplement: S1 File — (PDF) [file pone.0284682.s004.pdf]

ኮተቤ ሜትሮፖሊታን ዩኒቨርሲቲ
ዳግማዊ ምኒልክ ህክምናና ጤና ሳይንስ ኮሌጅ
ዲን ጽ/ቤት

KOTEBE METROPOLITAN UNIVERSITY
Menelik II Medical & Health Science College
Office of Dean

ቁጥር/Ref.No hms/38/12/2726
ቀን/Date: 28/07/2013

ለሰሜን ሼዋ ዞን ጤና ጽ/ቤት
ሰሜን ሸዋ ዞን፣

ጉዳዩ፡ የ Ethical clearance መስጠት ይመለከታል

ከላይ በርእሱ ለመግለፅ እንደተሞከረው የሁለተኛ ዲግሪ በPublic Health Nutrition ተመራቂ ተማሪ የሆነዉ ተማሪ ገብረፃድቅ ካለብ የመመረቅያውን ጥናት የሚትሰራበትን የጥናቱን ንድፈሐሳብ Ethical clearance ግምገማ እንድደረግ በዳግማዊ ሚኒልክ ህክምናና ጤና ሳይንስ ኮሌጅ፡ የምርምርና ህትመት አስተባባሪ ጽ/ቤት በድህረ ምረቃ ፕሮግራም አስተባባሪ ጽ/ቤት በቀን 28/07/139.ም ተጠይቆ ነበር፡፡ በጥያቄዉ መሰረት የኮሌጃችን የምርምርና ህትመት አስተባባሪ ጽ/ቤት "Undernutrition and associated factors among children aged 6-59 months in nutrition-sensetive agriculture implemented districts, North Shewa Zone, Ethiopia" የተባለሁን የተማሪዉ የመመረቅያ ጥናቱ ንድፈሐሳብ የEthical clearance ግምገማ የተደረገለት መሆኑን እያሳወቅን አስፈላጊዉን ትብብር እንዲደረግለት በአክብሮት እንጠይቃለን፡፡

ከሠላምታ ጋር!

ገመቹ አመዩ
[Stamp: ... ምርምርና ህ... / HEAD OF RESE...]

ግልባጭ፡-
- ለ ኮሌጁ ዲን
- ለ ትም/ም/ም/ም/ዲን
- ለ የምርምርና ህትመት አስተባባሪ
- ለ ድህረ ምረቃ ፕሮግራም አስተባባሪ
- ለ ገብረፃድቅ ካለብ

ዳ/ም/ህ/ጤ/ሳ/ኮሌጅ

ስልክ ቁጥር:
Tel. No. 0111 223828/27/26
ለዘላቂ የከተማ ልማት የሚተጋ!!
Committed to Sustainable Urban Development
አዲስ አበባ ኢትዮጵያ
Addis Ababa Ethiopia

Website: www.kmu.edu.et
