# Supplementary material for: Undernutrition and associated factors among children aged 6–59 months in nutrition-sensitive agriculture intervention implemented Basona district, North Shewa Zone, Amhara region, Ethiopia
Source: PLoS One. 2023 Apr 26;18(4):e0284682. doi: 10.1371/journal.pone.0284682 (PMC10132697; doi:10.1371/journal.pone.0284682)
Supplement: S2 File — (PDF) [file pone.0284682.s005.pdf]

ኢትዮጵያ ትርጉም ጽ/ቤት
ETHIOPIA TRANSLATION OFFICE
(091) 152-2689, (091) 250-5025
አዲስ አበባ፣ ኢትዮጵያ - ስታዲየም ህንፃ ቁ. 409 (ለ)
Addis Ababa, Ethiopia - Stadium Bldg. No. 409 (B)

Emblem

KOTEBE METROPOLITAN UNIVERSITY

Menelik II Medical and Health Science College

Office of Dean

Ref no: አመከ/38/12/2726

Date: 06/04/2021

To North Shewa Zone Health Office

North Shewa Zone

Subject: Regards Provision of Ethical Clearance

As tried to mention under the subject student Gebretsadik Keleb, graduating student in the field of Public health Nutrition with Masters degree, was requested by Menelik II Medical and Health Science College, Research and Print Coordination Office, Post-Graduate degree Program Coordination Office on date 06/04/2021 for the Ethical Clearance reviewing of his research topic. According to the request Our College's Research and Print Coordination Office announces that ethical Clearance review is done on the research topic of the student known as "Under-nutrition and associated factors among children and 6-59 months in nutrition -sensitive agriculture implemented districts, North Shewa Zone, Ethiopia"; we request that necessary support/cooperation shall be provided for him.

ኢትዮጵያ ትርጉም ጽ/ቤት
091 152 2689
አዲስ አበባ፣ ኢትዮጵያ
Addis Ababa, Ethiopia
ETHIOPIA TRANSLATION OFFICE

ኢትዮጵያ ዋለ
ዋና ሥራ አስኪያጅ
ETHIOPIA WALLE
General Manager

Kind regards

Signed

Gemechu Ameya

Head of Research and Print Coordination Office

<u>CC</u>

- To The Dean of the College
- To Educational System Registration and Reviewing Dean
- To Research and Print Coordination
- To Post-Graduation program Coordination
- To Gebretsadik Keleb

<u>Menelik II Medical and Health Science College</u>

<u>Seal</u>

KOTEBE METROPOLITAN UNIVERSITY

Menelik II Medical and Health Science College

Office of Dean

ኢትዮጵያ ትርጉም ጽ/ቤት
091 152 2689
አዲስ አበባ፣ ኢትዮጵያ
Addis Ababa, Ethiopia
ETHIOPIA TRANSLATION OFFICE

ኢትዮጵያ ዋለ
ዋና ሥራ አስኪያጅ
ETHIOPIA WALLE
General Manager

[illegible]
